# POLE Deficiency Exacerbates Diesel Engine Exhaust‐Induced Genomic Instability and Malignant Transformation of Bronchial Epithelial Cells

**DOI:** 10.1002/advs.202415943

**Published:** 2025-06-29

**Authors:** Pimei Zhang, Zhaoxu Wu, Qiang Ju, Nuo Xu, Xian Chen, Hongguang Chen, Shuaishuai Yang, Jing Ji, Yanjie Zhao

**Affiliations:** ^1^ Department of Blood Transfusion The Affiliated Hospital of Qingdao University Qingdao University Qingdao Shandong 266000 China; ^2^ School of Public Health Qingdao University Qingdao Shandong 266021 China

**Keywords:** DEE‐OEs, genomic instability, malignant transformation, POLE

## Abstract

Diesel engine exhaust (DEE) is classified as a Group I carcinogen, yet direct experimental evidence that DEE induce carcinogenesis is lacking. Here, it is innovatively discovered that 120 µg mL^−1^ DEE exposure for 20 weeks, original bronchial epithelial cells exhibit abnormal morphology, form colonies in soft agar, and readily develop the lumps under the subcutaneous tissue of immunodeficient mice, which are pathologically confirmed as lung squamous cell carcinoma. Whole genome sequencing and RNA sequencing identify DEE‐induced mutational signatures (DBS3, ID1/ID2), associated with polymerase epsilon (POLE) exonuclease domain mutations and mismatch repair (MMR) deficiency. Besides, 52 exonic mutations, 734 copy number variations (CNVs), and 2519 differentially expressed genes (DEGs) are discovered which are enriched in the suppressed DNA damage repair pathways and the activated lung cancer related signaling pathways including JAK‐STAT, PI3K‐Akt, MAPK in the DEE‐induced malignant transformed cells. Further assays confirm DEE‐induced malignant transformed cells harbor both POLE and MMR defects, leading to high mutation burden and genomic instability. Additionally, POLE deficiency exacerbates DEE‐induced DNA damage and genomic instability, promoting the cell malignant transformation. This study highlights the importance of gene‐environment interaction in carcinogenesis and emphasizes the critical role of POLE deficiency in DEE‐induced malignant transformation of lung cells.

## Graphical abstract

This study establishes a diesel engine exhaust (DEE)‐induced malignant transformation model in bronchial epithelial cells. Whole genome sequencing（WGS） and RNA‐seq reveal DEE‐induced mutational signatures and gene expression profiles. Mechanistically, DEEaffect polymerase epsilon (POLE) and mismatch repair, driving genomic instability and promoting malignant transformation. These emphasize the critical role of POLE and genetic‐environmental interactions in DEE‐induced carcinogenesis.

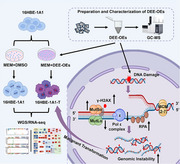

## 1 Introduction

Diesel engine exhaust (DEE) is one of the major air pollutants and has been classified as a “Group 1 carcinogen” by the International Agency for Research on Cancer.^[^ ^1^ ^]^ The main components of DEE include particulate matter, gaseous organic and inorganic substances, and nitro‐polycyclic aromatic hydrocarbons (nitro‐PAHs).^[^ ^2^ ^]^ These substances can enter the lungs through inhalation and spread throughout the body via the bloodstream, causing damage to various tissues.^[^ ^3^ ^]^ Epidemiological studies have shown a significant association between DEE exposure and increased lung cancer risk.^[^ ^4^ ^]^ However, there is still lack of experimental evidence proving that DEE exposure directly induces lung cancer.

Whole‐genome sequencing (WGS) enables comprehensive analysis of genomic alterations in cancer, aiding in the identification of driver mutations and their signatures.^[^ ^5^ ^]^ Somatic mutations in cancer are caused by multiple mechanisms, including errors during DNA replication, exposure to exogenous or endogenous mutagens, enzymatic modifications, and DNA repair deficiencies.^[^ ^6^ ^]^ Different mutational processes give rise to specific mutational signatures, which have been extensively cataloged in the Catalogue of Somatic Mutations in Cancer (COSMIC) database.^[^ ^7^ ^]^ Classification and analysis of these signatures allow for a deeper understanding of mutational mechanisms and their roles in cancer.^[^ ^6^ ^]^ For example, mutational signatures from DNA repair defects, like those in mismatch repair (MMR) (SBS6/SBS21/SBS26, ID1/ID2), show widespread mutation accumulation and are often linked with microsatellite instability (MSI).^[^ ^8^ ^]^ Environmental factors can also drive distinct mutational processes, as seen with APOBEC mutational signatures (SBS2/SBS13, DBS11), driven by APOBEC enzymes, have been found in malignant cells transformed by PM2.5 exposure, showing C > T and C > G changes.^[^ ^9^ ^]^ However, the mutational signatures associated with DEE exposure remain unclear.

POLE and MMR system are two key mechanisms that ensure the fidelity of genome replication.^[^ ^10^ ^]^ Mutations in the exonuclease domain of POLE result in loss of proofreading function, leading to mutation accumulation across the genome.^[^ ^11^ ^]^ Such mutations are found in various cancers, including endometrial cancer (EC), colorectal cancer (CRC), and lung cancer.^[^ ^12^ ^]^ When POLE proofreading function is impaired, the MMR system can usually correct some of the errors.^[^ ^13^ ^]^ However, dual deficiencies in POLE and MMR lead to an increased accumulation of mutations, resulting in hypermutator phenotypes with significant genomic instability.^[^ ^14^ ^]^ In EC, patients with coexisting POLE and MMR defects typically exhibit a high tumor mutation burden (TMB) and MSI status,^[^ ^14^, ^15^ ^]^ similar observations have been reported in CRC.^[^ ^14^, ^16^ ^]^

In this study, a malignant transformation model of bronchial epithelial cells was established through exposure to the organic extract of DEE (DEE‐OEs). WGS and RNA‐sequencing (RNA‐seq) analysis were conducted to investigate the genetic alterations and mutational signatures in the malignant transformation cells. Here we reported DEE‐OEs exposure led to POLE and MMR defects, cause to DNA damage and genomic instability, which promoting cell malignant transformation. This study highlights the critical role of POLE loss in DEE‐OEs‐induced malignant transformation and provides new insights into the carcinogenic mechanisms of DEE‐OEs exposure.

## 2 Results

### 2.1 DEE‐OEs Exposure Induces Transformation of Bronchial Epithelial Cells

**Figure 1:**
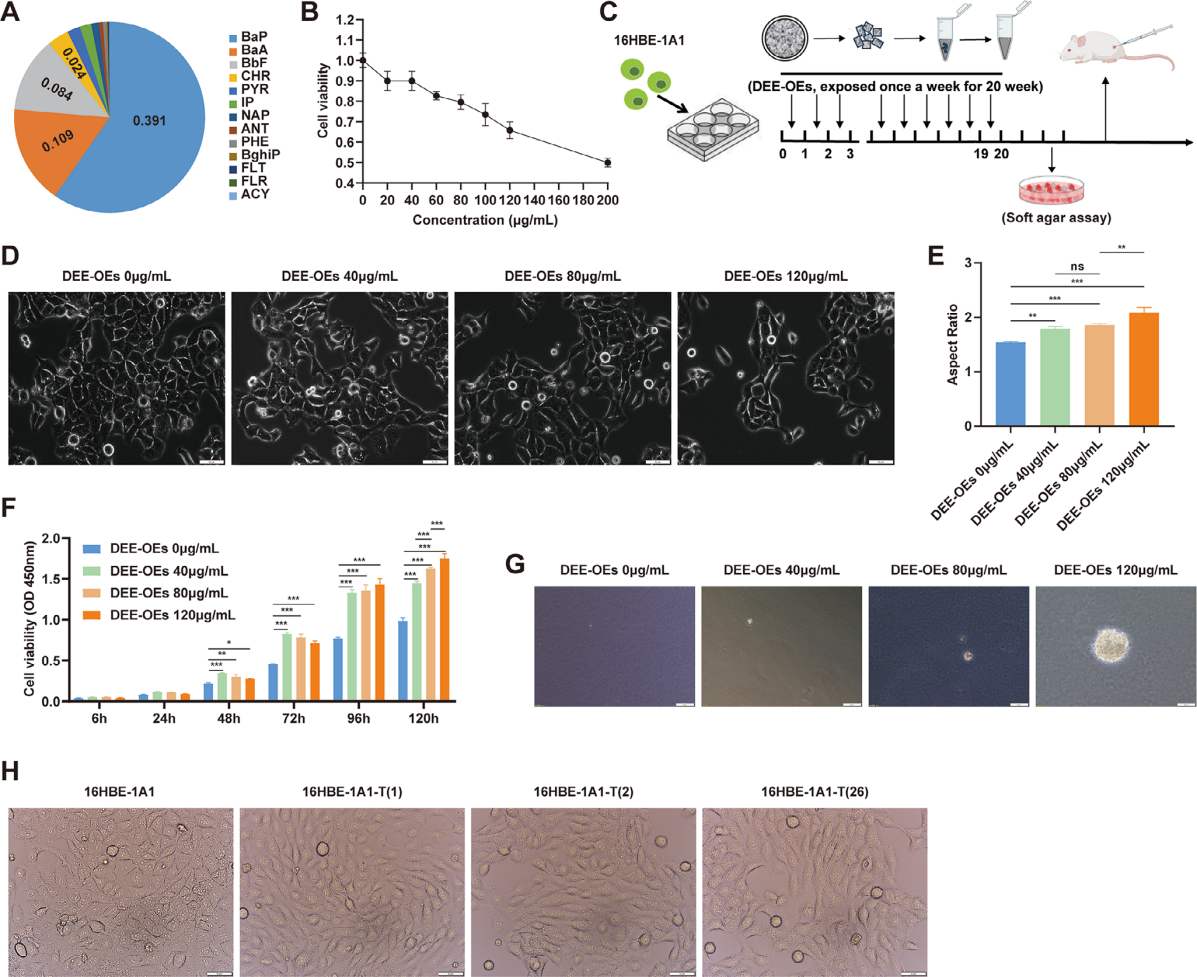
DEE‐OEs exposure induces malignant transformation of bronchial epithelial cells. (A) PAHs in DEE and their BaPeq, with the values in the figure representing the BaPeq of each component. Among these 16 PAHs, acenaphthene, benzo[k]fluoranthene and dibenzo[a,h]anthracene were not detected. (B) Dose‐response relationship of DEE‐OEs concentration and cell validity in 16HBE1A1 cells. (C) Schematic of the malignant transformation cell model induced by DEE‐OEs. (D,E) Cell morphology changes (D) and aspect ratio analysis (E) after 20 weeks of treatment with 0, 40, 80, and 120 µg mL^−1^ DEE‐OEs. Scale bar = 50 µm. (F) Relative proliferation levels of cells, which were treated with 0, 40, 80, and 120 µg mL^−1^ DEE‐OEs for 20 weeks, were measured by CCK‐8 assay. (G) Sphere formation with unanchored growth of DEE‐OEs‐induced transformation cells. (H) Cell morphology of three clonal cell lines including 16HBE‐1A1‐T(1), 16HBE‐1A1‐T(2), 16HBE‐1A1‐T(26), with 16HBE‐1A1 as the normal control. Scale bar = 50 µm. *p < 0.05, **p < 0.01, ***p < 0.001.

Polycyclic aromatic hydrocarbons (PAHs) are a family of toxicants with strong carcinogenicity.^[^ ^17^ ^]^ To assess the carcinogenic potential of DEE, we first characterize the concentrations of 16 major PAHs in DEE. Using these concentrations and the toxic equivalency factor (TEF) for each component, we calculated the Benzo[a]pyrene equivalent concentration (BaPeq) of DEE to be 0.654 µg m^−^^3^, with Benzo[a]pyrene (BaP), Benzo[a]anthracene (BaA), and Benzo[b]fluoranthene (BbF) being the primary contributors to BaPeq (**Figure** **1**A; and Table S1, Supporting Information).

To further investigate the carcinogenic potential of DEE, DEE‐OEs were extracted and used in the cell transformation assay. Based on the cytotoxicity detection, we determined the dose‐response relationship between DEE‐OEs concentration and cell validity in 16HBE‐1A1 cells (Figure 1B). DEE‐OEs exposure concentrations was selected as 0, 40, 80, and 120 µg mL^−1^ to conduct the cell transformation assay as outlined in Figure 1C. After continuous exposure for 20 weeks, significant morphological changes were observed. With the increase of exposure concentration, the cells gradually transitioned from a normal round shape to an elongated morphology (Figure 1D,E). To exclude the influence of cell density on morphology, we analyzed cell morphology at the same cell density. Results showed a consistent transition (Figure S1A,C, Supporting Information). Moreover, we collected cells at different time points (5, 10, 15, 20 W) and found with the exposure time increased, cells morphology in the 120 µg mL^−1^ group also gradually changed from a normal round shape to an elongated morphology (Figure S1B,D, Supporting Information). Interestingly, the cell growth rate is significantly accelerated with the increase of exposure concentration and exposure time (Figure 1F; and, Figure S1E, Supporting Information). After 20 weeks of DEE‐OEs exposure, cells were cultured without treatment for an additional 3 weeks. Interestingly, distinct cell colonies were formed in soft agar in the 120 µg mL^−1^ DEE‐OEs group, while did not form in the 40 and 80 µg mL^−1^ exposure groups (Figure 1G). Subsequently, we randomly selected three colonies including 16HBE‐1A1‐T(1), 16HBE‐1A1‐T(2), 16HBE‐1A1‐T(26) which formed on soft agar for expansion and culture. The expanded colonies exhibited pronounced size heterogeneity and abnormal morphology, with elongated shapes and filopodia‐like structures (Figure 1H). These results indicate that cells exposed to 120 µg mL^−1^ DEE‐OEs may exhibited malignant phenotypes.

### 2.2 Malignant Characteristics of the DEE‐OEs‐Induced Transformation Cell Model

**Figure 2:**
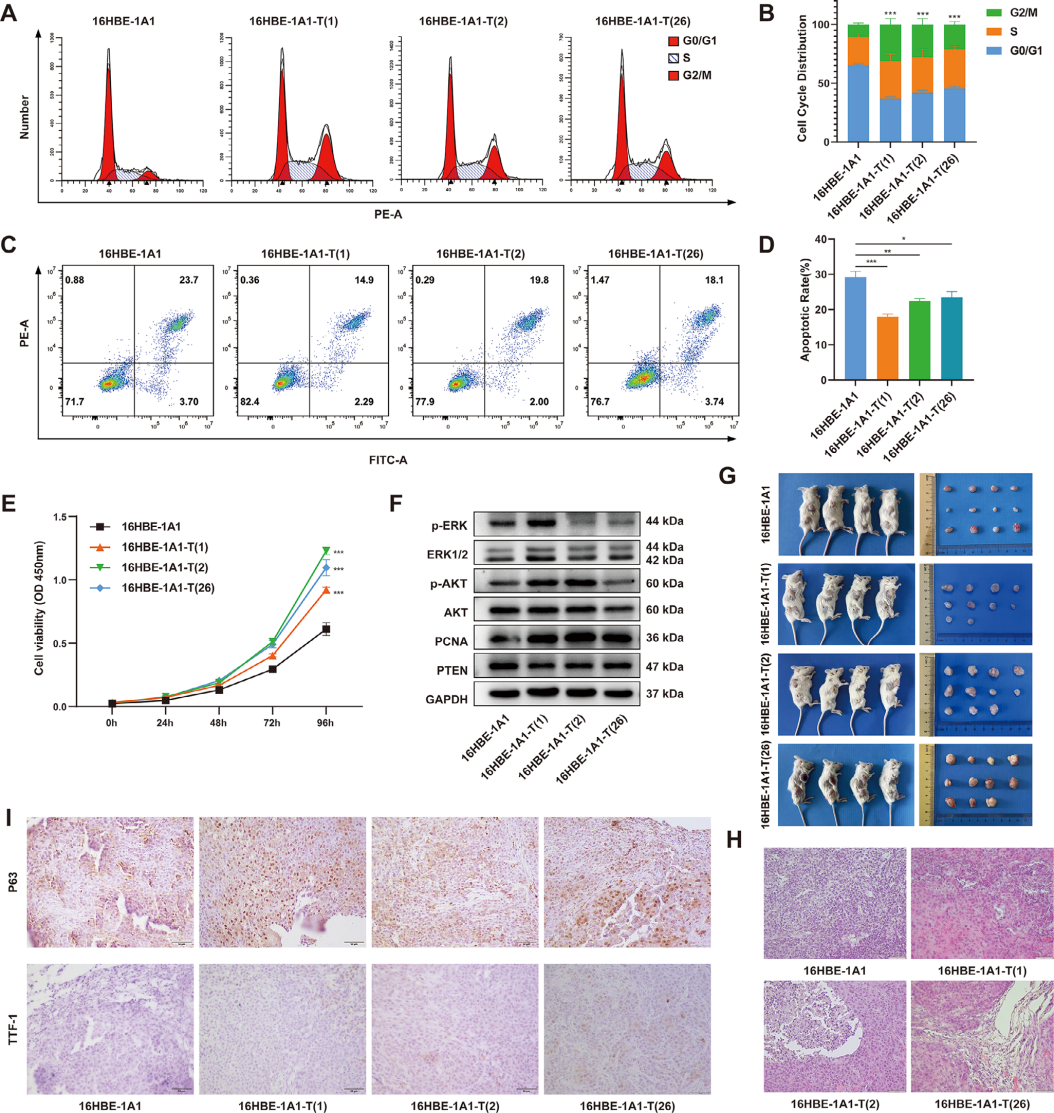
Malignant characteristics of the DEE‐OEs‐induced transformation cell model. (A–D) Flow cytometry analysis of cell cycle distribution (A) and cell apoptosis (C) in 16HBE‐1A1 cells and three clonal cell lines, with quantification of cell cycle phases (B) and Annexin‐FITC/PI positivity (D). (E) Relative proliferation levels of 16HBE‐1A1 cells and three clonal cell lines were measured by CCK‐8 assay. (F) Expression of lung cancer‐related proteins, including pERK/ERK, pAKT/AKT, PCNA, and PTEN in 16HBE‐1A1 cells and three clonal cell lines, was detected by Western blot analysis. (G) Lumps formed in the armpits or subcutis of NSG mice that were injected with 16HBE‐1A1 cells or the three clonal cell lines. *n* = 4 in all groups. (H) Representative H&E staining of xenografts derived from 16HBE‐1A1 cells and three clonal cell lines. Scale bar = 50 µm. (I) IHC analysis of P63 and TTF‐1 expression in xenografts derived from 16HBE‐1A1 cells and three clonal cell lines. Scale bar = 50 µm.

To identify the phenotypic characteristics of DEE‐OEs‐induced transformation cell models, we analyzed the cell cycle, cell apoptosis, cell proliferation in vitro and subcutaneous tumor formation in vivo. Compared with the control group, G1 phase was shortened, while S and G2 phases were significantly prolonged in the clonal cell lines (**Figure** **2**A,B). Additionally, cell apoptosis was reduced (Figure 2C,D), and proliferative capacity was enhanced (Figure 2E) in the transformation cells. Next, to investigate whether DEE‐OEs‐induced cell transformation resembles the process of lung tumorigenesis, we analyzed the expression of lung cancer‐related proteins, including PCNA, PTEN, AKT, and ERK pathways. Interestingly, results showed PCNA expression was upregulated and PTEN expression was downregulated, while AKT pathway was activated in all three clonal cell lines (Figure 2F; and Figure S2A–D, Supporting Information). However, ERK pathway activation was observed only in the 16HBE‐1A1‐T(1) clonal cell line due to the cell heterogeneity (Figure 2F; and Figure S2A–D, Supporting Information).

Furthermore, we assessed the tumorigenic potential of DEE‐OEs‐induced transformation cells in vivo. Results showed that all three clonal cell lines could form visible lumps in the armpits or subcutis of NSG mice (Figure 2G). Interestingly, xenografts derived from these clonal cell lines had significantly increased volume compared with the control group (Figure 2G). TUNEL staining showed very few positive staining was observed in transplanted tumors (Figure S2E, Supporting Information), suggesting that the observation of necrotic cavities in transplanted tumors may be caused by rapid tumor growth, not apoptosis. Hematoxylin and eosin (H&E) staining revealed these xenografts were arranged in nodular, sheet‐like, and trabecular patterns, with fibrosis observed in the interstitial spaces, and necrotic cavities formed in the central regions compared with the control (Figure 2H), further proved the malignant phenotype of DEE‐OEs‐induced transformed cell model. Moreover, to determine the tumor properties of malignant transformed cells, we performed immunohistochemical (IHC) using the lung squamous cell carcinoma (LUSC) marker P63 and the lung adenocarcinoma (LUAD) marker TTF‐1. Results showed that P63 was strongly positive, while TTF‐1 was negative in the xenografts derived from all three clonal cell lines (Figure 2I). These findings indicate that DEE‐OEs‐induced malignant transformation cells exhibit phenotypic characteristics of LUSC.

### 2.3 Genomic Alterations Induced by DEE‐OEs in the Process of Malignant Transformation

**Figure 3:**
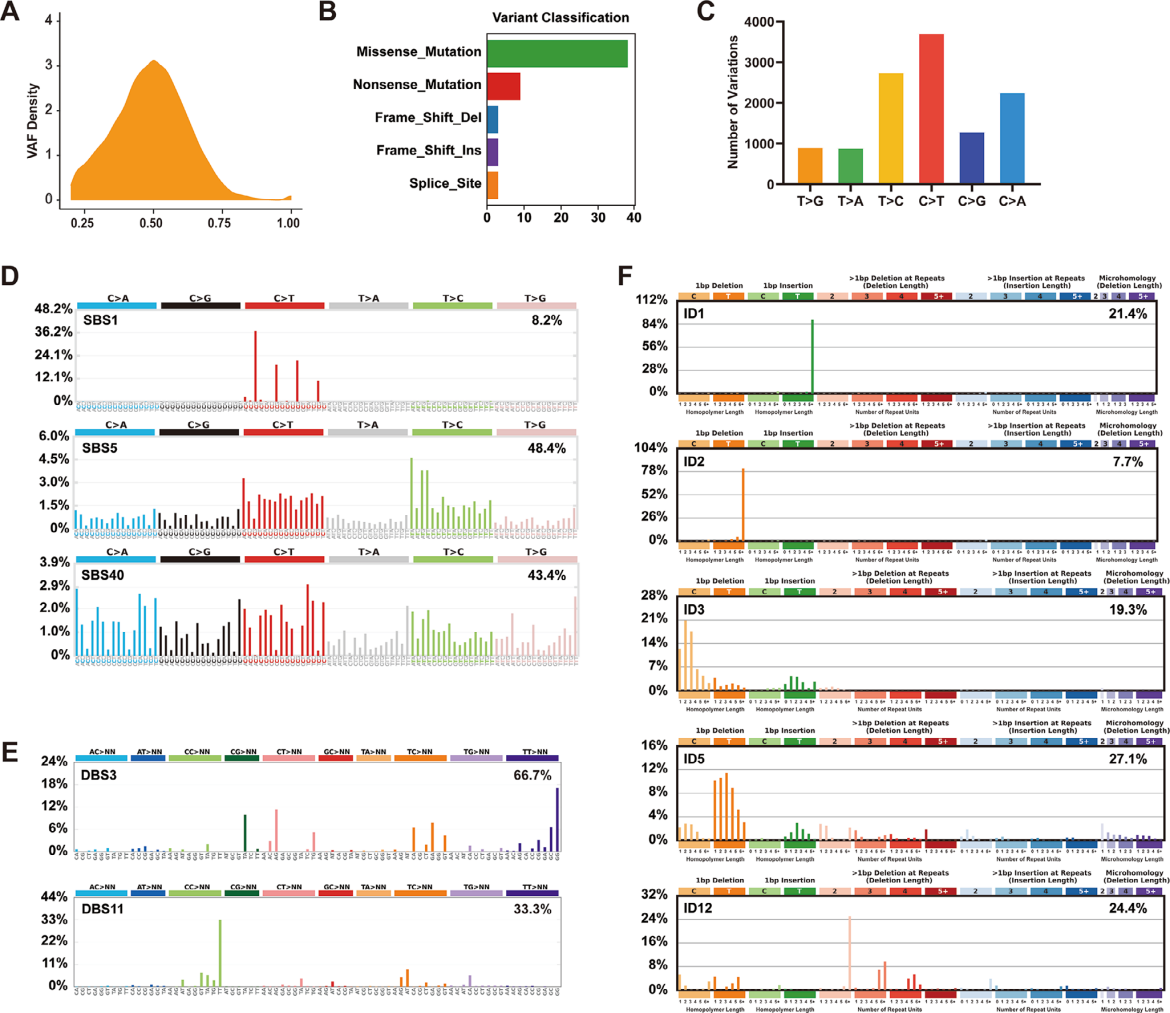
Somatic mutations and mutational signatures induced by DEE‐OEs exposure. (A) Density distribution of VAF in somatic mutations. (B) Proportional distribution of mutation types in somatic mutations. (C) Number of base substitution types in somatic mutations. (D–F) Catalogue of known COSMIC SBS/DBS/ID signatures detected in 16HBE‐1A1‐T cells. Signatures were displayed according to the 96/78/83‐substitution classification. X‐axes show mutation types, and Y‐axes show the frequency of each mutation type.

Gene alterations play a key role in driving cancer initiation and progression.^[^ ^18^ ^]^ To investigate the genomic variants associated with DEE‐OEs‐induced malignant transformation, WGS was performed based on the malignant transformed cells (16HBE‐1A1‐T) and normal 16HBE‐1A1 cell. 11 866 single nucleotide variations (SNVs) and 1130 insertions and deletions (InDels) were identified (Supplemental DateFile 1). The variant allele frequency (VAF) is primarily concentrated between 0.25 and 0.5 (**Figure** **3**A), and the mutations are mostly missense mutations (Figure 3B). The main types of base substitutions are T > C, C > T, and C > A (Figure 3C), accounting for 23.2%, 31.4%, 19.1% of all somatic SNVs, respectively. Of these mutations, we obtained 52 genes with SNVs or InDels in exon regions (Table S2, Supporting Information). To further verify the possible important roles of these altered genes in LUSC carcinogenesis, we analyzed the mutation frequency of these 52 genes in 492 LUSC cases from the TCGA database. Results showed that CSMD3 (47%), PAPPA2 (23%), MUC17 (21%), PCLO (21%), and RELN (19%) had high mutation frequencies (Figure S3A, Supporting Information). We selected these 5 genes for further validation, and all selected mutations were confirmed by PCR‐based Sanger sequencing (Figure S3B, Supporting Information). Furthermore, frequent mutations in these genes have been observed in other types of lung cancer (Figure S4A,B, Supporting Information), suggesting that these mutations in genes may drive malignant transformation following DEE‐OEs exposure. Additionally, we identified Kataegis sites on chromosome 7 (Figure S5, Supporting Information), which are localized hypermutations and often associated with genomic instability caused by pollutants exposure.^[^ ^19^ ^]^

### 2.4 Mutation Signatures Induced by DEE‐OEs Exposure in the Process of Malignant Transformation

Mutational signature analysis can reveal different carcinogenic mechanisms and defects in DNA repair processes by identifying and categorizing mutation patterns in cancer genome.^[^ ^6^ ^]^ Next, we analyzed these somatic mutations by applying SigProfiler. De novo decomposition results showed the single‐base substitutions (SBS) signatures induced by DEE‐OEs exposure are predominantly T > C, C > T, and C > A, accounting for 73.7% of all somatic SNVs (Figure S6A, Supporting Information). The double‐base substitutions (DBS) signatures are mainly TG > CA and TT > GG (Figure S6B, Supporting Information). The small insertions and deletions (ID) signatures primarily involve insertions or deletions of T within 1 bp (Figure S6C, Supporting Information). We then compared the mutational signatures in DEE‐OEs‐induced malignant transformation with COSMIC. The SBS were highly similar to three COSMIC signatures: SBS1 (8.2%), SBS5 (48.4%), and SBS40 (43.4%) (Figure 3D). SBS1 and SBS5 are associated with aging and are commonly found in various cancers, while the biological significance of SBS40 remains unclear, and its potential link to DEE‐OEs exposure requires further investigation. Additionally, two COSMIC signatures were identified from the DBS induced by DEE‐OEs exposure: DBS3 (66.7%), which is related to POLE exonuclease domain mutations that impair proofreading ability and lead to a high mutation rate, and DBS11 (33.3%), which may be associated with APOBEC mutagenesis (Figure 3E). The decomposition of InDels revealed signatures associated with MMR deficiency (ID1, 21.4%; ID2, 7.7%) and those related to smoking status (ID3, 19.3%). Additionally, ID5 and ID12 were observed, but their causes remain unknown (Figure 3F).

### 2.5 Copy Number Variations (CNVs) Drive Gene Expression Changes and Activate Lung Cancer‐Related Signaling Pathways

**Figure 4:**
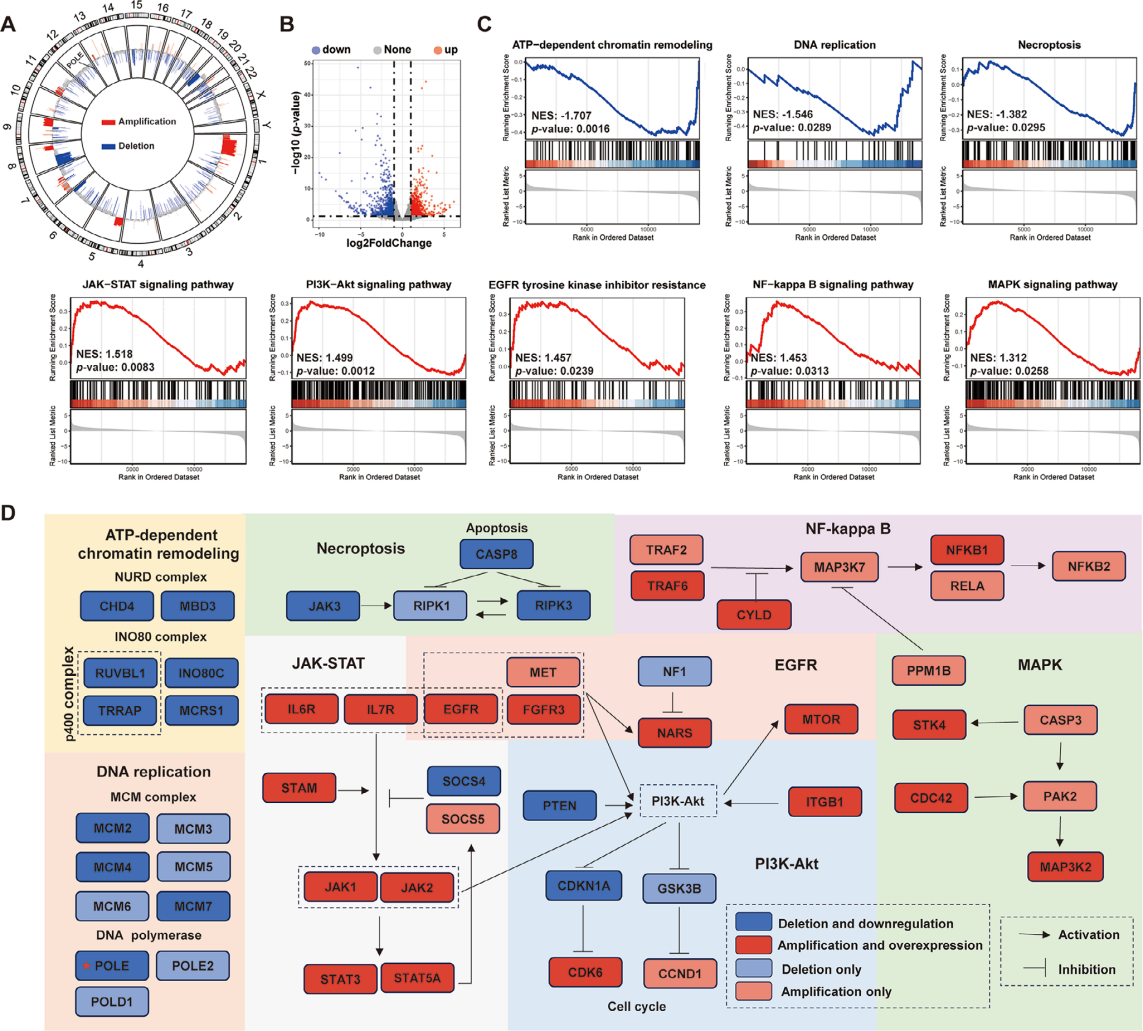
CNVs drive gene expression changes and activate lung cancer‐related signaling pathways. (A) Circos plot of CNVs: the outer ring represents chromosomes, while the inner ring shows CNVs, with red indicating amplifications and blue indicating deletions. (B) Volcano plot shows DEGs between 16HBE‐1A1‐T and 16HBE‐1A1 cells. (C) GSEA showing the enrichment of DNA damage repair pathways and lung cancer‐related pathways in 16HBE‐1A1‐T compared to 16HBE‐1A1 cells. (D) Pathways and networks related to DNA damage repair and lung cancer: different colored rectangles represent gene statuses identified in 16HBE‐1A1‐T cells, including amplification, amplification with overexpression, deletion, and deletion with low expression. Amplification and deletion were determined by CNVkit. Overexpression and low expression were defined by Student's *t*‐test between 16HBE‐1A1 and 16HBE‐1A1‐T cells, with *p* < 0.05 and expression fold change > 1.2 or < 0.8 considered significant.

Next, we identified 734 CNVs, predominantly located in specific chromosomal arms, including amplifications in 1p, 5p, 7p, 8q, 9q, and 11p, as well as deletions in 6q, 8p, and 20p (**Figure** **4**A). Notably, we observed a deletion of POLE among these CNVs (Figure 4A). Given that CNVs can directly influence gene expression,^[^ ^20^ ^]^ we conducted RNA‐seq analysis on 16HBE‐1A1 and 16HBE‐1A1‐T cells. Differential expression analysis identified 2519 differentially expressed genes (DEGs), including 1352 upregulated genes and 1167 downregulated genes in 16HBE‐1A1‐T cells (Figure 4B; and Supplemental DateFile 2). GSEA enrichment analysis revealed suppression of DNA repair pathways, such as ATP‐dependent chromatin remodeling, DNA replication, as well as the necroptosis pathway (Figure 4C). In contrast, classical lung cancer‐related pathways, including the JAK‐STAT signaling pathway, PI3K‐Akt signaling pathway, EGFR tyrosine kinase inhibitor resistance, NF‐kappa B signaling pathway, and MAPK signaling pathway, were significantly activated (Figure 4C; and Table S3, Supporting Information).

Combining CNVs analysis, we identified significant copy number losses and downregulation of key genes (such as CHD4, RUVBL1, INO80C, and MCRS1) within the NuRD, INO80, and p400 complexes, which may severely impair their functions in chromatin remodeling and DNA damage repair.^[^ ^21^ ^]^ Additionally, several key genes associated with the MCM complex and DNA polymerase pathways, including MCM2, MCM4, MCM7, and POLE, also exhibited losses and downregulation, further indicating significant defects in DNA repair mechanisms.^[^ ^11^, ^22^ ^]^ These defects may lead to increased genomic instability, thereby promoting carcinogenesis (Figure 4D; and Figure S7, Supporting Information). In terms of signaling pathways, various oncogenes in the PI3K‐Akt pathway, such as EGFR and FGFR3, showed amplification and overexpression, indicating pathway activation that promotes cell proliferation and survival. The overactivity of the PI3K‐Akt pathway is particularly common in various cancers, especially lung cancer.^[^ ^23^ ^]^ Furthermore, the activation of the NF‐kappa B and MAPK pathways, evidenced by the amplification of genes such as TRAF6, and MAP3K2, promotes the expression of antioxidant genes, as well as cell proliferation and survival.^[^ ^24^ ^]^ Moreover, in the JAK‐STAT pathway, key genes (including JAK1, JAK2, and STAT3) also displayed amplification, suggesting this signaling pathway may play a crucial role in cancer cell growth and immune evasion.^[^ ^25^ ^]^ Concurrently, tumor suppressor genes related to cell cycle regulation, such as PTEN and CDKN1A, exhibited losses and downregulation, inhibiting normal cell cycle control and driving uncontrolled cell proliferation (Figure 4D; and Figure S7, Supporting Information).^[^ ^26^ ^]^

Overall, the integrated analysis of CNVs and RNA‐seq highlights the significant loss and downregulation of key genes associated with chromatin remodeling, DNA damage repair, and cell cycle regulation in tumor cells, leading to defective DNA repair mechanisms and increased genomic instability. Additionally, the amplification and overexpression of oncogenes in the PI3K‐Akt, NF‐kappa B, MAPK, and JAK‐STAT pathways suggest that their activation promotes cell proliferation and immune evasion. This is consistent with the high expression of ERK and AKT observed in Figure 2F, highlighting the activation of these pathways in malignant transformation. These results emphasize the complex molecular changes induced by DEE‐OEs in malignant transformation and their importance in cancer progression.

### 2.6 Malignant Transformation Cells Induced by DEE‐OEs Exhibit Characteristics of POLE Loss, MMR Deficiency, and Genomic Instability

**Figure 5:**
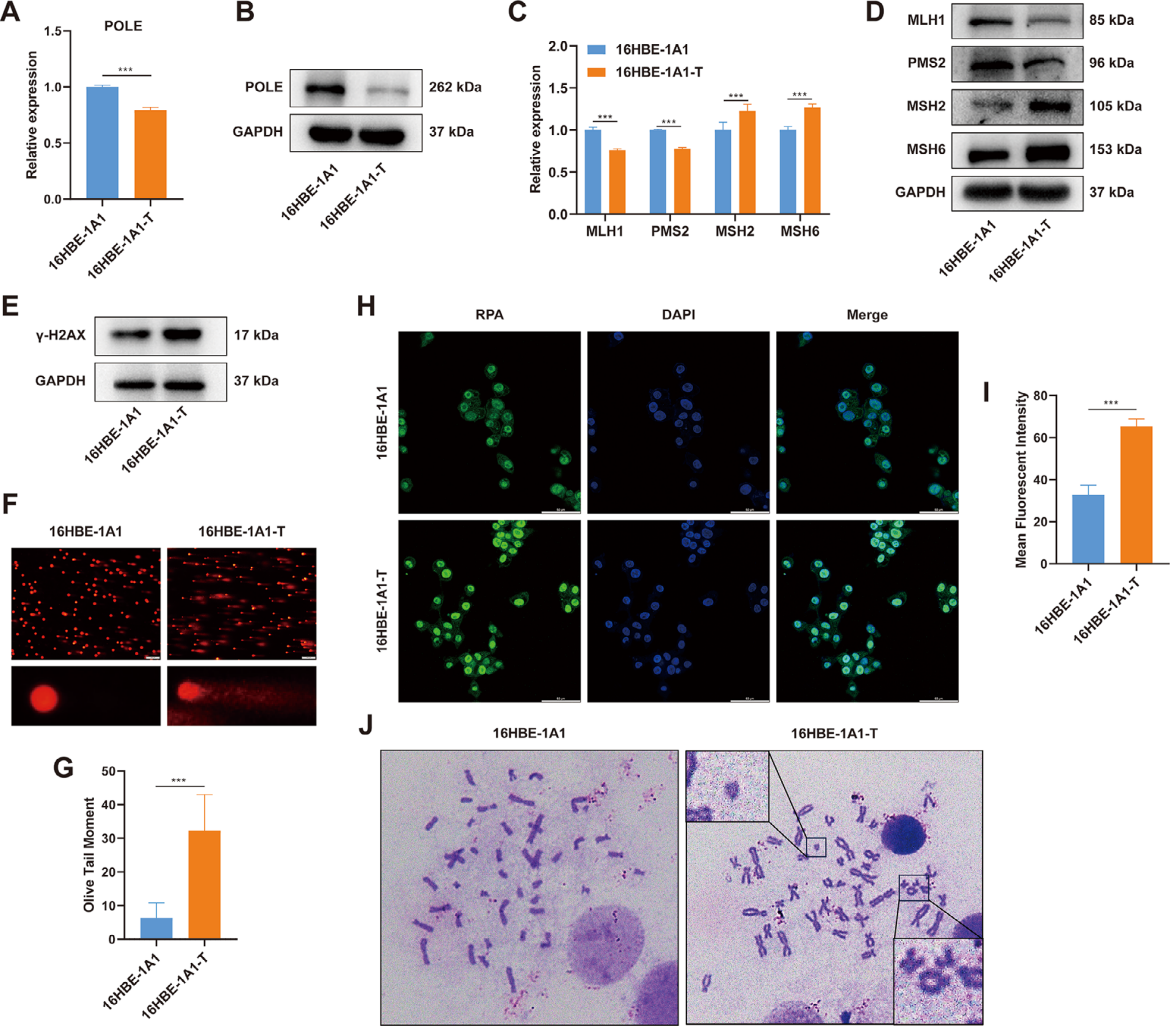
Malignant transformation cells induced by DEE‐OEs exhibit characteristics of POLE and MMR deficiency, and genomic instability. (A,B) mRNA (A) and protein (B) expression levels of POLE in the 16HBE‐1A1 and 16HBE‐1A1‐T cells. (C,D) mRNA (C) and protein (D) levels of the key enzymes (MLH1, PMS2, MSH2, MSH6) of the MMR pathway in the 16HBE‐1A1 and 16HBE‐1A1‐T cells. (E) Expression levels of γ‐H2AX protein in the 16HBE‐1A1 and 16HBE‐1A1‐T cells. (F,G) Comet assay analysis of DNA damage levels in the 16HBE‐1A1 and 16HBE‐1A1‐T cells, scale bar = 200 µm. (H, I) Representative images (H) and quantitative analysis of average fluorescence intensity (I) from RAP immunofluorescence staining in the 16HBE‐1A1 and 16HBE‐1A1‐T cells, scale bar = 50 µm. (J) Chromosome spreading analysis in the 16HBE‐1A1 and 16HBE‐1A1‐T cells, with aberrations magnified and annotated. *p < 0.05, **p < 0.01, ***p < 0.001.

To further elucidate the impact of DEE‐OEs exposure on DNA repair pathways, we conducted validation of DNA repair‐related genes and pathways involved in the observed mutation signatures (DBS3, ID1/ID2). Results show that the mRNA and protein levels of POLE are significantly downregulated in 16HBE‐1A1‐T cells (**Figure** **5**A,B; and Figure S8A, Supporting Information). Kaplan–Meier survival analysis further reveals a significant association between low POLE expression and poor prognosis in LUSC (Figure S8B, Supporting Information). Moreover, we performed a correlation analysis using RNA‐seq data and revealed associations between POLE expression and several key driver genes in LUSC (Figure S8C, Supporting Information). Furthermore, we observed MMR deficiencies in 16HBE‐1A1‐T cells, characterized by upregulation of MutSα (MSH2‐MSH6), and downregulation of MutLα (MLH1‐PMS2) (Figure 5C,D; and Figure S8D–G, Supporting Information). These findings suggest that DEE‐OEs exposure may impair DNA repair capacity to induce cell malignant transformation.

The decline in DNA repair capacity may contribute to genomic instability.^[^ ^27^ ^]^ Therefore, we further assessed DNA damage and chromosomal aberrations in both 16HBE‐1A1 and 16HBE‐1A1‐T cells. Results revealed a significant increase in γ‐H2AX protein expression in 16HBE‐1A1‐T compared with 16HBE‐1A1 (Figure 5E; and Figure S8H, Supporting Information), suggesting that the DNA damage response had been activated by DEE‐OEs exposure. Alkaline comet assays showed an increase in olive tail moment (OTM) in 16HBE‐1A1‐T compared with 16HBE‐1A1 (Figure 5F,G), reflecting higher levels of DNA breaks. Considering that single‐stranded DNA is protected by RPA protein during nascent strand degradation, we analyzed RPA foci formation in both 16HBE‐1A1 cells and 16HBE‐1A1‐T cells. Results showed a significant increase in RPA foci in 16HBE‐1A1‐T cells (Figure 5H,I), indicating substantial replication stress. In addition, chromosomal abnormalities, including breaks and ring structures, were observed in 16HBE‐1A1‐T cells (Figure 5J). Overall, these results indicate that DEE‐OEs exposure leads to POLE loss and MMR deficiency, impairs DNA repair capacity, thereby causing DNA damage accumulation and genomic instability to induce cell malignant transformation.

### 2.7 POLE Loss Exacerbates DEE‐OEs Exposure‐Induced Genomic Instability

**Figure 6:**
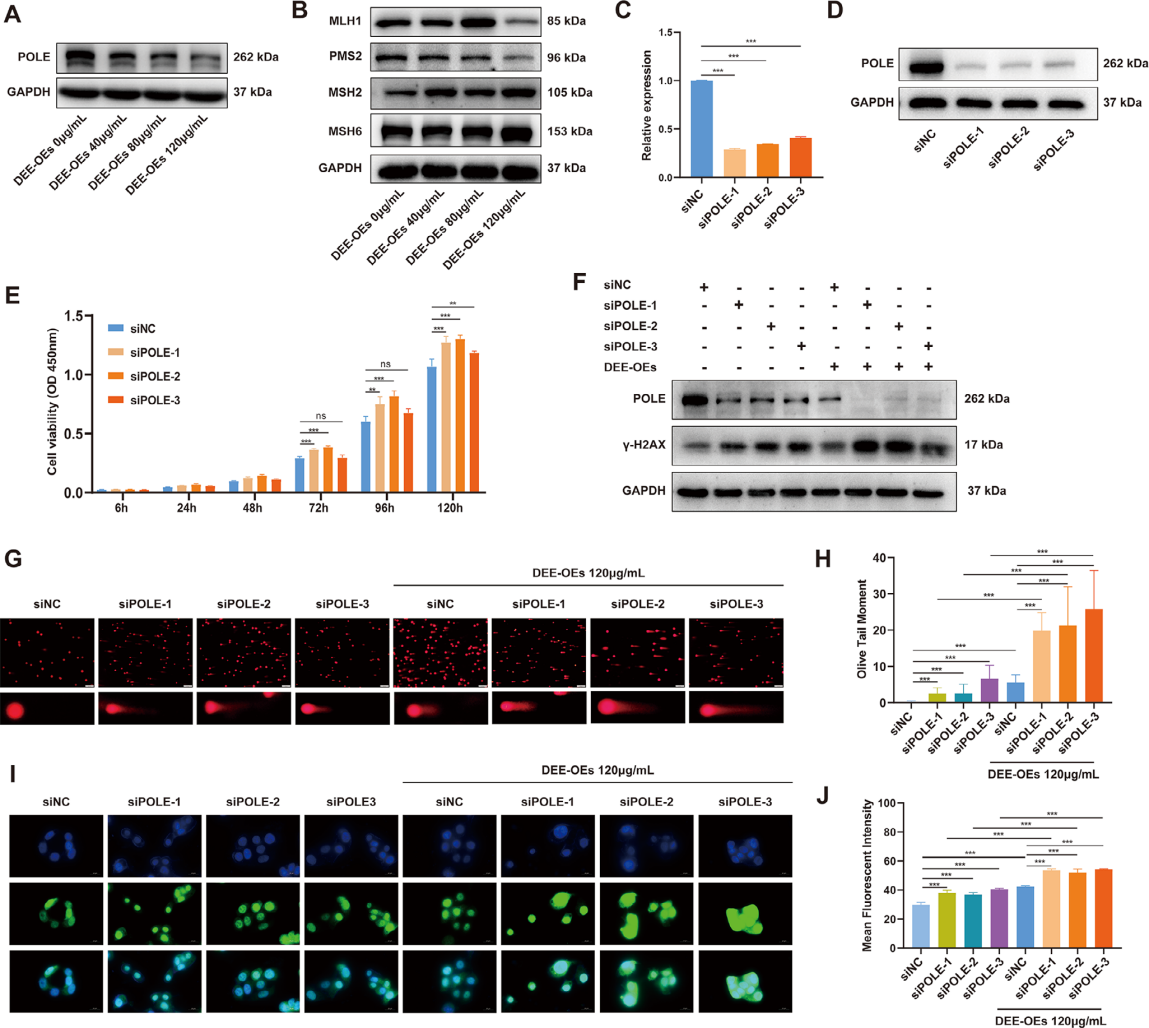
POLE loss combined with DEE‐OEs exposure exacerbates genomic instability. (A,B) The expression levels of POLE protein (A) and key genes in the MMR pathway (MLH1, PMS2, MSH2, MSH6) (B) were assessed through Western blot after 20 weeks of exposure to 0, 40, 80, and 120 µg mL^−1^ DEE‐OEs. (C,D) The mRNA and protein expression of POLE following siRNA‐mediated knockdown. (E) The relative proliferation levels of 16HBE‐1A1 cells subjected to POLE knockdown was measured using the CCK‐8 assay. (F) Expression levels of γ‐H2AX protein in 16HBE‐1A1 cells after POLE knockdown, DEE‐OEs exposure, and combined treatment. (G,H) DNA damage levels in 16HBE‐1A1 cells subjected to POLE knockdown, DEE‐OEs exposure, and their combined treatment were assessed using the comet assay (G), along with quantitative analysis of the OTM (H), scale bar = 200 µm. (I,J) Representative images of RAP immunofluorescence in 16HBE‐1A1 cells after POLE knockdown, DEE‐OEs exposure, and their combined treatment (I), along with quantitative analysis of the mean fluorescence intensity (J), scale bar = 20 µm. *p < 0.05, **p < 0.01, ***p < 0.001.

To investigate whether POLE loss and MMR deficiency are driving events in malignant transformation, we assessed 16HBE‐1A1 cells after 20 weeks of DEE‐OEs exposure at grade concentrations (0, 40, 80, 120 µg mL^−1^). Results showed a dose‐dependent decrease in POLE protein levels with increasing DEE‐OEs exposure (**Figure** **6**A; and Figure S9A, Supporting Information). However, MutSα protein levels exhibited a slight increase at 40 and 80 µg mL^−1^ DEE‐OEs, with a significant increase observed at 120 µg mL^−1^. Meanwhile, MutLα protein levels did not change significantly at 40 and 80 µg mL^−1^ but significantly decreased at 120 µg mL^−1^, suggesting that DEE‐OEs exposure affects the MMR pathway indirectly (Figure 6B; and Figure S9B–E, Supporting Information). Given the critical role of POLE in DNA repair, we hypothesize that the DEE‐OEs‐induced downregulation of POLE expression may play a significant role in driving the malignant transformation of 16HBE‐1A1 cells. Next, we knocked down the POLE expression in 16HBE‐1A1 cells (Figure 6C,D; and Figure S9F, Supporting Information) and found the decrease of POLE significantly promoted cell growth (Figure 6E). Furthermore, we explored the relationship between POLE loss and the MMR pathway after knockdown and found no direct correlation (Figure S9G, Supporting Information). γ‐H2AX protein levels was elevated based on POLE downregulation (Figure 6F; and Figure S9H,I, Supporting Information). However, DEE‐OEs exposure resulted in a more significantly increase in γ‐H2AX expression induced by POLE downregulation, indicating an exacerbation of DNA damage accumulation (Figure 6F; and Figure S9H,I, Supporting Information). Alkaline comet assay results showed that POLE knockdown or DEE‐OEs exposure alone increased the OTM, with a more significantly effect when DEE‐OEs exposure based on POLE knockdown, indicating enhanced DNA breakage (Figure 6G,H). Similarly, both POLE knockdown or DEE‐OEs exposure alone increased RPA foci, and DEE‐OEs exposure based on POLE knockdown further increased RPA foci formation, suggesting increased replication stress (Figure 6I,J). These results indicate that POLE loss exacerbates DEE‐OEs exposure‐induced DNA damage and genomic instability.

### 2.8 POLE Overexpression Restores DEE‐OEs‐Induced Cell Phenotype and Genomic Instability

**Figure 7:**
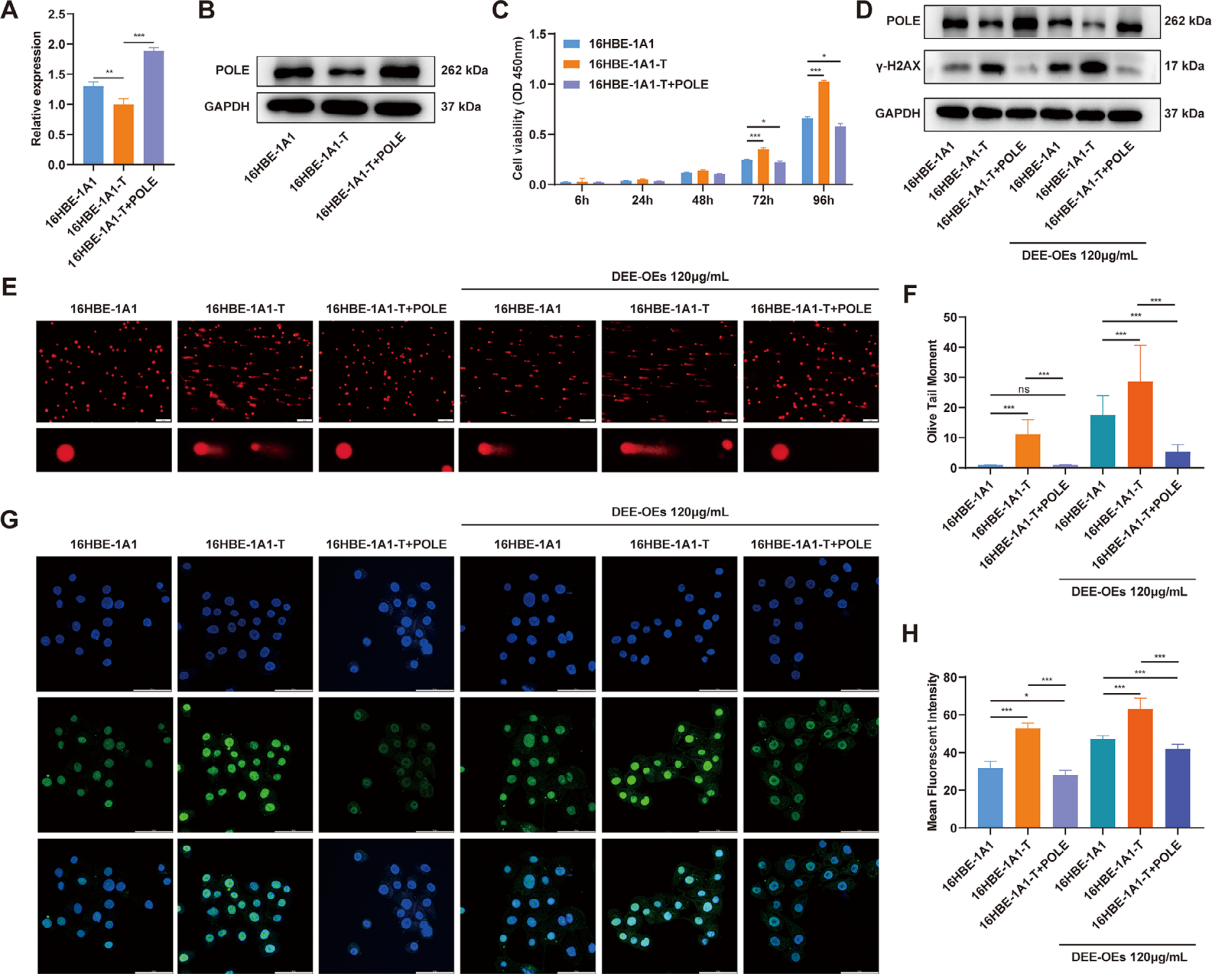
POLE overexpression restores DEE‐OEs‐induced cell phenotype and genomic instability. (A,B) The mRNA (A) and protein (B) expression levels of POLE in 16HBE‐1A1‐T cells overexpressing POLE. (C) Relative proliferation levels of 16HBE‐1A1‐T cells subjected to POLE overexpression, measured using the CCK‐8 assay. (D) Expression levels of γ‐H2AX protein in 16HBE‐1A1‐T cells after POLE overexpression, DEE‐OEs exposure, and combined treatment. (E,F) DNA damage levels in 16HBE‐1A1‐T cells subjected to POLE overexpression, DEE‐OEs exposure, and their combined treatment, assessed using the comet assay (E), along with quantitative analysis of the OTM (F), scale bar = 100 µm. (G,H) Representative images of RAP immunofluorescence in 16HBE‐1A1‐T cells after POLE overexpression, DEE‐OEs exposure, and their combined treatment (G), along with quantitative analysis of the mean fluorescence intensity (H), scale bar = 50 µm. *p < 0.05, **p < 0.01, ***p < 0.001.

To further verify the significant role of POLE in DEE‐OEs‐induced malignant transformation, we overexpressed the POLE expression in 16HBE‐T cells (**Figure** **7**A,B; and Figure S10A, Supporting Information) and found POLE overexpression significantly inhibited cell growth of 16HBE‐1A1‐T (Figure 7C). Moreover, γ‐H2AX protein levels were significantly decreased in 16HBE‐T cells with POLE overexpression. Interestingly, DEE‐OEs exposure resulted in a significant decrease of γ‐H2AX protein induced by POLE overexpression (Figure 7D; and Figure S10B,C, Supporting Information). In addition, we performed Alkaline comet assay and RPA foci detection assay to investigate the effect of POLE overexpression on the genomic instability. Results showed POLE overexpression in 16HBE‐T cells could restore the increase of OTM (Figure 7E,F) and RPA foci (Figure 7G,H) regardless of DEE‐OEs exposure. All these suggest POLE overexpression could restore DEE‐OEs‐induced cell phenotype and genomic instability, further verified the significant role of POLE loss in DEE‐OEs‐induced malignant transformation.

## 3 Discussion

In this study, we first established a malignant transformation model of bronchial epithelial cell induced by DEE‐OEs exposure and identified DEE‐OEs‐induced mutational signatures (DBS3, ID1/ID2). Moreover, we revealed significant inhibition of certain DNA damage repair pathways, alongside notable activation of lung cancer‐related signaling pathways. Furthermore, this study innovatively discovered the presence of both POLE and MMR deficiencies in DEE‐OEs‐induced malignant transformation cells, while POLE deficiency further exacerbating DEE‐OEs‐induced DNA damage and genomic instability. These results strongly indicate the lung carcinogenicity of DEE exposure and POLE deficiency may be the key point leading to the malignant transformation of bronchial epithelial cell.

DEE is the common air pollutant whose toxic components primarily originate from PAHs present in the organic phase.^[^ ^28^ ^]^ PAHs are significant environmental carcinogens, which could induce metabolic activation to form reactive intermediates, DNA adduct formation, gene mutations, and disruption of cell signaling pathways.^[^ ^29^ ^]^ These processes may promote abnormal cell proliferation and transformation, thereby increasing cancer risk.^[^ ^30^ ^]^ PAHs are widely present in air pollutants, such as PM2.5 and COE. Previous study has demonstrated that emissions from steel production can induce malignant transformation in normal bronchial epithelial cells, thereby elevating the lung cancer risk among coke oven workers.^[^ ^31^ ^]^ Chen et al. revealed that long‐term exposure to PM2.5 leads to malignant transformation of bronchial epithelial cells.^[^ ^32^ ^]^ Additionally, PM2.5 significantly accelerate lung cancer development in mice.^[^ ^33^ ^]^ All these findings indicate that DEE rich in PAHs may induce malignant transformation of lung cells. However, traditional animal models are more suited to acute toxicity studies. In vivo carcinogenicity tests are time‐consuming, costly, and require large numbers of animals, limiting their utility for investigating mechanisms of lung cancer induced by chronic exposure.^[^ ^34^ ^]^ Therefore, we established a malignant transformation model using human bronchial epithelial cells to serve as a more efficient alternative for evaluating DEE's carcinogenic potential. In the present study, we have experimentally demonstrated for the first time that bronchial epithelial cell 16HBE‐1A1 exhibit distinct malignant characteristics after 20 weeks of DEE‐OEs exposure, including morphological changes, enhanced clonogenic ability in soft agar, and the histological features of lung squamous cell carcinoma in subcutaneous xenografts. These results experimentally validate previous studies that squamous cell carcinoma is the lung cancer subtype most strongly associated with DEE exposure.^[^ ^35^ ^]^ Moreover, 16HBE cell line used in this study is derived from human bronchial epithelial cells, which are the primary origin of lung squamous cell carcinoma. These findings strongly support the notion that DEE‐OEs possess carcinogenic potential similar to other PAHs pollutants, capable of inducing malignant transformation.

Tumorigenesis is the result of somatic mutations of genetic material.^[^ ^36^ ^]^ Mutational signatures reflect the roles of different mutational processes in cancer development, thereby providing insights into the potential carcinogenic factors or mechanisms responsible for genomic alterations.^[^ ^36^, ^37^ ^]^ In the present study, we found mutations induced by DEE‐OEs exposure exhibit notable similarities to those characterized in smoking‐related lung cancer.^[^ ^38^ ^]^ Specifically, both smoking‐related lung cancer and DEE‐OEs exposure are characterized by SBS1 and SBS5 signatures, which are often associated with endogenous mutagenesis or defects in DNA repair mechanisms.^[^ ^39^ ^]^ Additionally, we also found DBS associated with the APOBEC enzyme family,^[^ ^40^ ^]^ as well as mutational signatures linked to MMR defects (ID1 and ID2).^[^ ^39^, ^41^ ^]^ ID3 signature is also frequently observed in smoking‐related lung cancer patients, further indicating a shared carcinogenic mechanism between tobacco smoke and DEE.^[^ ^42^ ^]^ However, DEE‐OEs‐induced malignant transformation cells also displayed unique mutational features distinct from smoking‐related lung cancer. Notably, our study is the first to identify the DBS3 signature in DEE‐OEs‐induced malignant transformation cells, which is closely associated with POLE exonuclease domain mutations.^[^ ^43^ ^]^ This suggests that the toxic components of DEE may induce additional mutations via mechanisms distinct from smoking, thereby accelerating carcinogenesis. Furthermore, the study found that DEE‐OEs exposure leads to a dose‐dependent decrease in POLE expression, with higher DEE‐OEs concentrations associated with more pronounced malignant characteristics. Additionally, we examined the expression of POLE in LUSC patients and normal tissues in the TCGA database, and found no evidence of POLE deletion in LUSC patients. This suggests that POLE loss is not specific to LUSC but rather acts as a key mechanism in DEE‐induced malignant transformation. Overall, these results strongly indicate that POLE deficiency plays a critical role in DEE‐OEs‐induced malignant transformation, providing important insights into the mechanisms underlying DEE‐related cancer.

The proofreading function of DNA polymerase and MMR system are two essential mechanisms that ensure high‐fidelity of DNA replication to maintain genomic stability.^[^ ^10^ ^]^ POLE, as a subunit of DNA polymerase, primarily facilitates the synthesis of new strands during DNA replication and possesses proofreading capabilities to identify and correct base pairing errors that occur during replication.^[^ ^44^ ^]^ MMR pathway further ensures replication accuracy by recognizing and repairing mismatched base pairs.^[^ ^45^ ^]^ When POLE's proofreading function is impaired, the MMR pathway typically serves as a compensatory mechanism to repair errors that POLE cannot correct.^[^ ^13^, ^46^ ^]^ Previous studies found dual defects in POLE proofreading function and the MMR pathway are often associated with higher mutation burdens and increased MSI in CRC and EC.^[^ ^12^, ^14^ ^]^ In nonsmall cell lung cancer (NSCLC), POLE mutations correlate with TMB, PD‐L1 expression, and increased CD^8+^ tumor‐infiltrating lymphocytes.^[^ ^47^ ^]^ Moreover, POLE mutations in other solid tumors, such as gastric, esophageal, and ovarian cancers, display features of immune tolerance and defective mismatch repair, highlighting their potential as biomarkers for immunotherapy.^[^ ^48^ ^]^ Therefore, defects in either mechanism can lead to the accumulation of mutations, resulting in genomic instability and an increased risk of carcinogenesis.^[^ ^49^ ^]^ In the DEE‐OEs‐induced malignant transformation cells, both POLE deficiency and MMR pathway abnormalities were observed, resulted in increased DNA damage and genomic instability. Interestingly, MMR defects were only present in the 120 µg mL^−1^ DEE‐OEs exposure group, whereas POLE expression decreased in a dose‐dependent manner with increasing exposure. This suggests that DEE‐OEs may indirectly affect the MMR pathway while directly suppress POLE expression. Additionally, knockdown of POLE significantly exacerbated DNA damage and genomic instability induced by DEE‐OEs exposure. All these results suggested that POLE deficiency impairs the repair of DEE‐OEs‐induced DNA damage, leading to the accumulation of mutations, further aggravating genomic instability.

Genomic instability is a hallmark of cancer, typically associated with mutation accumulation, chromosomal rearrangements, and defects in DNA repair mechanisms, which collectively increase the likelihood of oncogene activation and tumor suppressor gene inactivation, ultimately promoting tumorigenesis.^[^ ^50^ ^]^ In the DEE‐OEs‐induced malignant transformation cells, we observed downregulation of tumor suppressor genes such as PTEN, CASP8, and CDKN1A, along with significant activation of oncogenes including EGFR, JAK, and STAT3. These alterations disrupt normal cell cycle regulation, DNA damage repair, and apoptosis, endowing cells with sustained proliferative and antiapoptotic capacities. Additionally, mutations driven by genomic instability can aberrantly activate cancer‐related signaling pathways, such as the PI3K/AKT, MAPK, and NF‐kappa B pathways, further promoting cellular proliferation and survival. As a crucial regulator of genomic stability, mutations in the POLE gene have been implicated in accelerating tumor progression through potential impacts on the MMR pathway, TGF‐β signaling, and the RTK/RAS/RAF pathway.^[^ ^48^ ^]^ In DEE‐OEs‐induced malignant transformation cells, MMR pathway abnormalities and activation of classic lung cancer‐related signaling pathways were indeed observed; these changes are closely associated with POLE deficiency‐induced genomic instability, forming a vicious cycle that further drives tumor development. Although the exact causal relationships of these mechanisms require further investigation, the potential link between POLE and key oncogenic pathways underscores its important role in tumorigenesis and offers new therapeutic targets and strategies for lung cancer and other malignancies. In recent years, POLE as a potential target for the treatment of lung cancer has attracted more and more attention. Studies have reported that lung cancer patients with POLE mutation showed a better response to immunotherapy, which may be related to the high tumor mutation burden caused by POLE mutations.^[^ ^51^ ^]^ Another study reported that POLE mutations can serve as independent biomarkers for predicting the efficacy of immunotherapy across multiple cancer types, providing more precise guidance for the clinical application of immunotherapy.^[^ ^52^ ^]^ Although POLE mutations are associated with favorable responses to immunotherapy, their specific application as therapeutic targets still face many challenges. For example, the frequency and type of POLE mutations vary among different lung cancer types, and their relationship with other gene mutations and clinical features is not fully understood. Therefore, more large‐scale clinical trials and in‐depth research are needed in the future to further validate the effectiveness and safety of POLE as a therapeutic target for lung cancer.

In conclusion, this study not only established for the first time that DEE‐OEs‐induced malignant transformation model of bronchial epithelial cells but also revealed the critical role of POLE deficiency in DEE‐OEs‐induced genomic instability and malignant transformation. These findings provide new insights into the carcinogenic mechanisms of air pollutants and offer important theoretical evidence for the prevention and treatment of lung cancer.

Nevertheless, our study has limitations. First, we selected colonies formed in soft agar for subsequent analyses. Although this approach effectively enriches for cells that have undergone stable malignant transformation and exhibit tumor‐like growth properties, it may inadvertently exclude cells in the early or intermediate stages of transformation. As a result, the full spectrum of cellular heterogeneity and the dynamic progression of the transformation process may not be fully captured. We will use the original treated cells to conduct phenotype assays to offer a more accurate representation of the transformation process and tumorigenesis. Second, the compositional analysis of DEE identified BaP as the most toxic constituent. However, the specific contribution of this compound to the overall carcinogenicity of DEE remains to be confirmed. Future studies should incorporate a control exposure group devoid of BaP to determine whether it holds the highest toxicity level among the compounds in the mixture. These investigations will provide a more refined understanding of the carcinogenic mechanisms underlying DEE exposure and offer a stronger scientific foundation for public health risk assessments.

## 4 Experimental Section

### Preparation and Characterization of DEE‐OEs

DEE was collected at a designated point using a particulate sampler (Tianhong, China). Polytetrafluoroethylene (PTFE) membrane (PALL, US) was employed to capture DEE particles and organic pollutants at a flow rate of 100 L min^−1^ for a duration of 10 min. Filter membranes were immersed in a solvent mixture of n‐hexane and dichloromethane (v/v = 2:1) for 48 h, followed by Soxhlet extraction to obtain organic extracts. The extracts were filtered through the 0.45 µm filter, dried under nitrogen flow, and dissolved in dimethyl sulfoxide.^[^ ^53^ ^]^ The types and concentrations of PAHs in the DEE were analyzed using gas chromatography‐mass spectrometry (GC‐MS) (Agilent Technologies Inc., USA). BaPeq were calculated by multiplying the concentration of PAHs with the corresponding TEF of individual PAH component.^[^ ^54^ ^]^

### Cell Culture and Treatments

Metabolic activation catalyzed by CYP1A1 is critical for the carcinogenicity of carcinogenic compounds.^[^ ^55^ ^]^ Due to the low expression of CYP1A1 in the wild‐type 16HBE cells, 16HBE cells were chosen stably transfected with CYP1A1.^[^ ^32^ ^]^ Human bronchial epithelial cell line 16HBE‐1A1 (stably over‐expressed CYP1A1) were generously provided by Professor Wen Chen. Cells were cultured in Minimum Essential Medium (MEM, BasalMedia, China) supplemented with 10% fetal bovine serum (FBS, Biological Industries, Israel) and 1% penicillin‐streptomycin (Gibco, USA) in a 37 °C, 5% CO_2_ humidified incubator. Multiple Path Particle Dosimetry (MPPD) computational model was used to estimate the deposition fraction of airborne DEE in the lung epithelium. Based on the prediction of the MPPD software, physiological parameters were used to determine the in vitro equivalent dose as described previously.^[^ ^56^ ^]^ The following equation was used

(1) $$DE=\frac{AB}{SA}\times C\times f\times \frac{PSA}{V}$$

*DE*, the in vitro equivalent dose corresponding to daily exposure to DEE; *SA*, the surface area of the lung epithelium (75 m^2^); AB, the breathing rate (18.42 m^3^ day^−1^); *C*, the concentration of ambient DEE (1000 µg m^−3^); *f*, the deposition fraction of airborne DEE in the lung epithelium predicted by MPPD software (0.37); *PSA*, the surface area per well (0.32 cm^2^); *V*, the medium volume per well (100 µL).

Through the above calculations, the in vitro equivalent dose corresponding to actual environmental exposure level is 291 µg mL^−1^ was obtained. Next, 291 µg mL^−1^ as the maximum allowable dose was identified for cytotoxicity experiments and designed the following dose: 0, 20, 40, 60, 80, 100, 120, 200 µg mL^−1^ to conduct cytotoxicity assay. Consider the three outcomes of cells after exposed with toxicant: cell death (dead cells), successful cell repair (normal cells), failure cell repair (mutational cells), concentration (120 µg mL^−1^) was set up with cell viability is more than 60% as the highest concentration for the cell transformation assay. Different concentrations of DEE‐OEs (0, 40, 80, 120 µg mL^−1^) were used to expose 16HBE‐1A1 cells weekly for a duration of 20 weeks. Subsequently, soft agar assays were performed every 2 weeks, and the resulting cell colonies were amplified for further culture.

### Determination of Cell Proliferation

Cells were inoculated in 96‐well plates at a density of 1 × 10^3^ cells per well. At specified time points (6, 24, 48, 72, 96, and 120 h), Cell Counting Kit‐8 reagent (Dojindo, Japan) was added. After incubating at 37 °C for 2 h, the absorbance at 450 nm for each well was measured using a multifunctional microplate reader (BioTek, USA).

### Soft Agar Assay

Anchorage‐independent cell growth was detected using a soft agar assay. 1 × 10^3^ cells were seeded in a 12‐well plate and cultured in the MEM medium plus 10% FBS in 0.4% agar above a layer of 0.8% agar. After 14 days, clone formation was observed under the microscope.

### Flow Cytometry Analysis of Cell Cycle and Apoptosis

The Cell Cycle and Apoptosis Kit (Bioscience, China) was used to assess cell cycle distribution. Cells were fixed with 70% ethanol overnight at 4 °C and stained with propidium iodide (PI) and RNase A. Cell cycle distribution was analyzed using a Beckman flow cytometer (Beckman, USA) and ModFit 5.0 software. For apoptosis detection, an Apoptosis Detection Kit (Yeasen, China) was employed. Cells were stained with Annexin V‐FITC and PI, and apoptosis rates were measured using the Beckman flow cytometer (Beckman, USA) and analyzed with FlowJo (v10.8.1) software. FITC^−^/PI^−^ cells were classified as live cells, FITC^+^/PI^−^ cells as early apoptotic, and FITC^+^/PI^+^ cells as late apoptotic or dead.

### Western Blot Analysis

Total protein was extracted by RIPA (Beyotime, China) and separated by SDS‐PAGE. Then proteins were transferred to a polyvinylidene fluoride (PVDF) membrane (Millipore, USA). The membrane was blocked with 5% nonfat milk and incubated overnight at 4 °C with primary antibodies: ERK (zenbio, China; 1:1000), pERK (zenbio, China; 1:1000), AKT (zenbio, China; 1:1000), pAKT (zenbio, China; 1:1000), PCNA (Santa, USA; 1:1000), PTEN (Santa, USA; 1:1000), POLE (GeneTex, USA; 1:1000), MSH2 (zenbio, China; 1:1000), MSH6 (zenbio, China; 1:1000), MLH1 (zenbio, China; 1:1000), PMS2 (zenbio, China; 1:1000), γ‐H2AX (zenbio, China; 1:500), and GAPDH (zenbio, China; 1:1000). Next day, membrane was incubated with goat antirabbit or goat antimouse secondary antibody and developed with ECL detection reagent (Oriscience, China). Images were captured using the Tanon 5200 (Tanon, China) and analyzed with ImageJ software.

### Subcutaneous Tumor Model

All animal experiments were approved by the Ethics Committee Medical College of Qingdao University (No: QDU‐AEC‐2024037) and carried out in compliance with the Animal Research Reporting of In‐Vivo Experiments (ARRIVE) guidelines. 16HBE‐1A1 and three clonal cells (16HBE‐1A1‐T (1), 16HBE‐1A1‐T (2), 16HBE‐1A1‐T (26)) suspension (1.2 × 10⁷ cells in 100 µL PBS) were subcutaneously injected into the two axillae and the right groin of the mice. Four weeks postinjection, mice were humanely euthanized in accordance with humane treatment policies for tumor‐bearing animals. Tumors were stripped and fixed with 4% paraformaldehyde for further histopathological analysis.

### H&E Staining

The fixed xenograft tissues were dehydrated and embed in paraffin to prepare 6 µm sections for histopathological examination. Then, sections were dewaxed, rehydrated, and stained with H&E (Beyotime, China). Images were captured using a microscope (Olympus, Japan).

### IHC

The sections were dewaxed and rehydrated, followed by antigen retrieval using citrate buffer (Solarbio, China). Samples were incubated with 3% H₂O₂, then blocked with 5% BSA. Primary antibodies, P63 (zenbio, China, 1:50) and TTF‐1 (zenbio, China, 1:50), were applied and incubated overnight at 4 °C. Then, sections were incubated with goat antirabbit IgG secondary antibody (Boster, China) at 37 °C. Signal detection was performed using a DAB substrate kit (Beyotime, China) and hematoxylin. Images were captured using the microscope (Olympus, Japan).

### Genomic Mutation Detection and Analysis

WGS of 16HBE‐1A1 and 16HBE‐1A1‐T (1) cells was performed on the Illumina platform. FastQC (v0.12.1) was used to assess the quality of the sequencing reads. Complete sequencing data quality control information were provided in Table S4 (Supporting Information). Reads were mapped to the GRCh38 genome using the default parameters of BWA mem (v0.7.17) and sorted using Samtools (v1.17). Subsequently, the Genome Analysis Toolkit (GATK v4.1.5.0) was utilized with default parameters to mark duplicates, perform local realignment, and recalibrate the BWA‐aligned reads.

SNVs and InDels were detected using Mutect2 in GATK, retaining mutations with the VAF > 0.2 and the sequencing depth > 10, followed by annotation using Annovar (Sun, 7 Jun 2020). The maftools R package was employed to create a rain plot of somatic mutations and a waterfall plot of exonic mutations. Mutation frequency of 52 identified exonic mutated genes was analyzed in 492 TCGA‐LUSC cases, with the top five genes subjected to PCR‐based Sanger sequencing. PCR primers were designed using Primer5 to amplify regions of 300–500 bp encompassing each mutation. The primer sequences are provided in Table S5, Supporting Information. Mutation signatures were identified using SigProfiler (v1.1.21), and cosine similarity was used to measure the similarity between the identified signatures and COSMIC signatures.

CNVs were identified using the default parameters of CNVkit (https://cnvkit.readthedocs.io), where the segment mean (seg.mean) > 0.25 indicates copy number amplification and seg.mean <−0.25 indicates copy number deletion. Visualization was performed using the circlize R package.

### RNA‐seq Analysis

Paired‐end sequencing of 16HBE‐1A1 and 16HBE‐1A1‐T (1) cells was performed on the Illumina NovaSeq platform, with three samples per group. FastQC (v0.12.1) was used to assess the quality of the sequencing reads. Complete sequencing data quality control information were provided in Table S6 (Supporting Information). The reads were mapped to GRCh38 using the default parameters of HISAT2 (v2.1.0), and gene count values were obtained using HTseq. DEGs between 16HBE‐1A1 and 16HBE‐1A1‐T cells were analyzed using DESeq2, applying the criteria of |log2FoldChange| > 1 and *p* < 0.05 for selection.

### RNA Isolation and Quantitative Polymerase Chain Reaction (qPCR)

Total RNA was extracted using Trizol reagent (Invitrogen, USA). Reverse transcription was performed with the Evo M‐MLV Reverse Transcription Premix Kit (Accurate Biology, China). Quantitative real‐time PCR (qRT‐PCR) was conducted using the SYBR Green Pro Taq HS Premix Kit (Accurate Biology, China), with GAPDH serving as the internal control. The relative expression of mRNA was calculated using the 2*^─^* ^ΔΔCt^ method. Primers were synthesized by Tsingke Biotechnology (Tsingke, China), and their sequences are detailed in Table S7, Supporting Information.

### Comet Assay

Cell suspension was mixed with 0.7% low‐melting agarose at 37 °C and dripped onto slides precoated with 1% normal‐melting agarose. Then, cells were lysed at 4 °C with lysis buffer and unwound in electrophoresis solution, followed by electrophoresis at 25 V under dim light. Staining was performed with PI solution, and images were captured using a fluorescence microscope (Eclipse Ci, Nikon). Quantitative analysis was conducted using CASP software.

### Chromosome Spread Analysis

Chromosome spread analysis was performed according to the description.^[^ ^57^ ^]^ Briefly, cells were incubated with 0.08 µg mL^−1^ colchicine for 4 h, followed by swelling in 0.075 m KCl at 37 °C. Subsequently, fixation was carried out using the 3:1 mixture of methanol and acetic acid, after which the suspension was dropped onto pre‐cooled slides and allowed to air dry at room temperature. Cells were stained with 10% Giemsa. Images were captured using the microscope (Olympus, Japan). A minimum of 50 cells were counted to assess chromosomal abnormalities.

### Immunofluorescence Analysis

Cells were fixed with 4% paraformaldehyde and permeabilized using 1% Triton X‐100, followed by blocking with 3% BSA. Slides were then incubated overnight at 4 °C with the primary antibody (RPA, zenbio, China, 1:50). Next day, samples were incubated with a fluorescent secondary antibody, and nuclei were counterstained with DAPI (Solarbio, China, 1:200). Immunofluorescence images were acquired using a Leica SP8 confocal microscope (Leica, Germany).

### Statistical Analysis

Experimental data were analyzed and plotted using GraphPad Prism 8.0. Results are presented as mean ± SEM (*n* ≥ 3 biological replicates). Comparisons between two groups were performed using Student's *t*‐test, while multiple group comparisons were conducted using one‐way ANOVA. *p* < 0.05 was considered to indicate statistical significance.

## Conflict of Interest

The authors declare no conflict of interest.

## Author Contributions

P.Z., Z.W., and Q.J. contributed equally to this work. P.Z., Z.W., Q.J., and Y.Z. designed the study and completed manuscript writing, review, and/or revision. P.Z. performed WGS and RNA‐seq data analysis and conducted the experiments. Z.W. constructed the malignant transformed cell model. N.X., X.C., H.C., S.Y., and J.J. provided helpful discussion. Q.J. and Y.Z. provided funding acquisition. All authors reviewed and edited the manuscript.

## Supporting information



Supporting Information

Supplemental DateFile 1

Supplemental DateFile 2

## Data Availability

The data that support the findings of this study are available from the corresponding author upon reasonable request.
