# Supplementary material for: POLE Deficiency Exacerbates Diesel Engine Exhaust‐Induced Genomic Instability and Malignant Transformation of Bronchial Epithelial Cells
Source: Adv Sci (Weinh). 2025 Jun 29;12(36):e15943. doi: 10.1002/advs.202415943 (PMC12463065; doi:10.1002/advs.202415943)
Supplement: Supplementary file 1 — Supporting Information [file ADVS-12-e15943-s003.docx]

**Supporting Information**

**POLE Deficiency Exacerbates Diesel Engine Exhaust induced Genomic Instability and Malignant Transformation of Bronchial Epithelial Cells**

Pimei Zhang ^1,2^, Zhaoxu Wu ^1,2^, Qiang Ju^1,^ ^2^, Nuo Xu ^2^, Xian Chen ^2^, Hongguang Chen ^2^, Shuaishuai Yang ^2^, Jing Ji ^2^, Yanjie Zhao ^2,^*

**Supplementary Material**

**This supporting information includes:**

**Figure S1:**

Changes in cell morphology and proliferative capacity during DEE-OEs exposure.

**Figure S2:**

Effects of DEE-OEs-induced transformation cells on lung cancer-related protein expression and TUNEL staining in xenografts.

**Figure S3:**

Mutation landscape of 52 exon-region mutant genes induced by DEE-OEs exposure in TCGA-LUSC.

**Figure S4.**

Mutation landscape of 52 exon-region mutant genes induced by DEE-OEs exposure in other lung cancer.

**Figure S5:**

Localized hypermutation rainfall plot induced by DEE-OEs exposure.

**Figure S6:**

De novo decomposition of mutational signatures in 16HBE-1A1-T cells.

**Figure S7:**

DNA damage repair and lung cancer-related signaling pathways were verified by qPCR.

**Figure S8:**

DEE-OEs-induced malignant transformation cells exhibit POLE deficiency, MMR defects, and increased DNA damage.

**Figure S9:**

Effects of DEE-OEs exposure and POLE knockdown on protein expression and DNA damage.

**Figure S10.**

Effects of DEE-OEs exposure and POLE overexpression on the protein expression of γ-H2AX.

**Table S1:**

Major components and concentrations of PAHs in DEE.

**Table S2:**

Somatic mutations in exonic regions identified by WGS.

**Table S3:**

Statistically significant pathways.

**Table S4.**

WGS sequencing data quality assessment.

**Table S5:**

Primers used for PCR.

**Table S6.**

RNA-Seq sequencing data quality assessment.

**Table S7:**

Primers used for quantitative qRT-PCR.


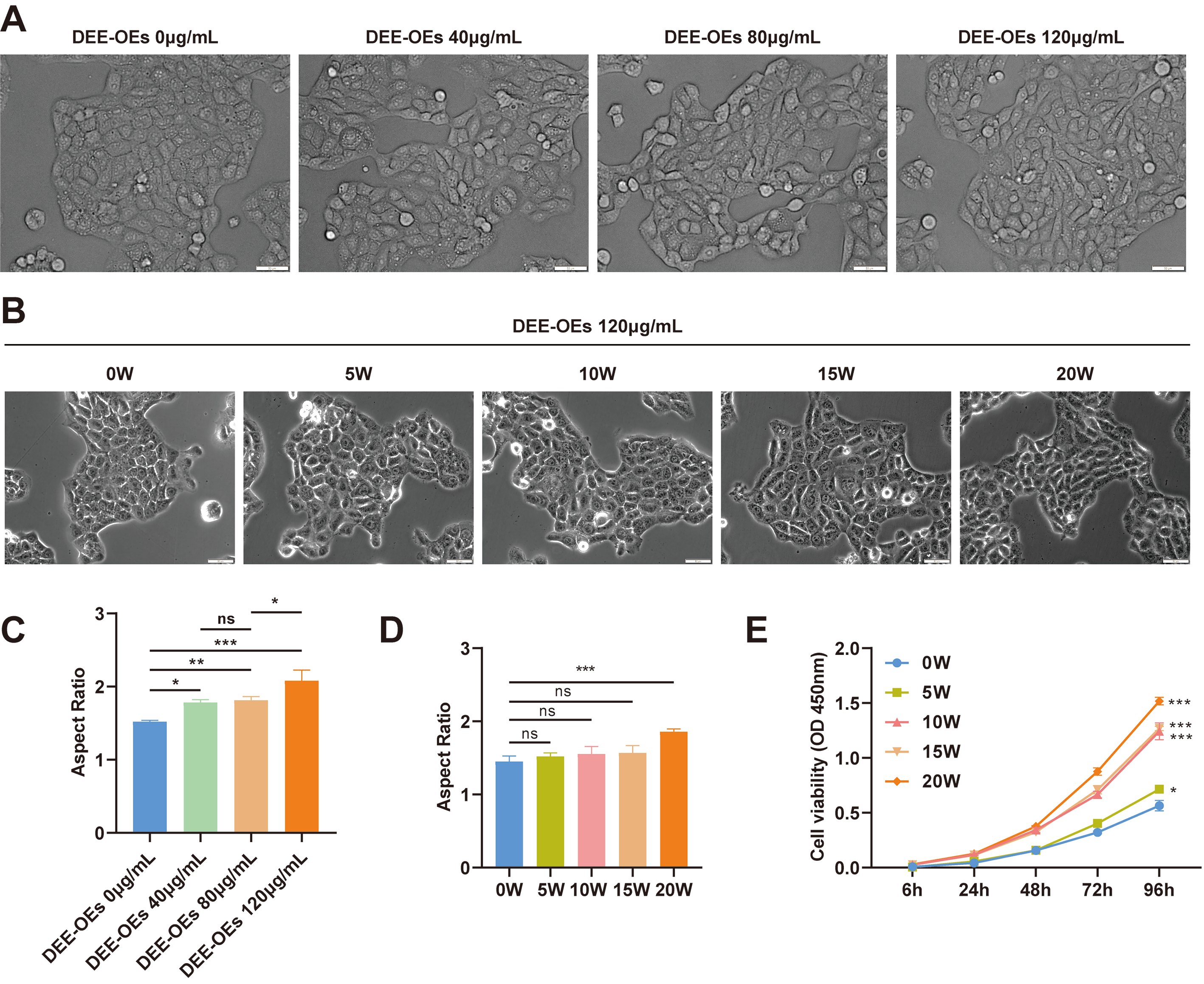


**Figure S1.** **Changes in cell morphology and proliferative capacity during DEE-OEs exposure. A.** Cell morphology changes after 20 weeks of treatment with 0, 40, 80, and 120 μg/mL DEE-OEs at the same cell density. Scale bar = 50 μM. **B.** Temporal changes in cell morphology with 120 μg/mL DEE-OEs at different time points (5W, 10W, 15W, 20W). Scale bar = 50 μM. **C.** Aspect ratio analysis after 20 weeks of treatment with 0, 40, 80, and 120 μg/mL DEE-OEs. D. Aspect ratio analysis with 120 μg/mL DEE-OEs at different time points (5W, 10W, 15W, 20W). E. Relative proliferation levels of cells treated with 120 μg/mL DEE-OEs at different time points (5W, 10W, 15W, 20W), as measured by CCK-8 assay.


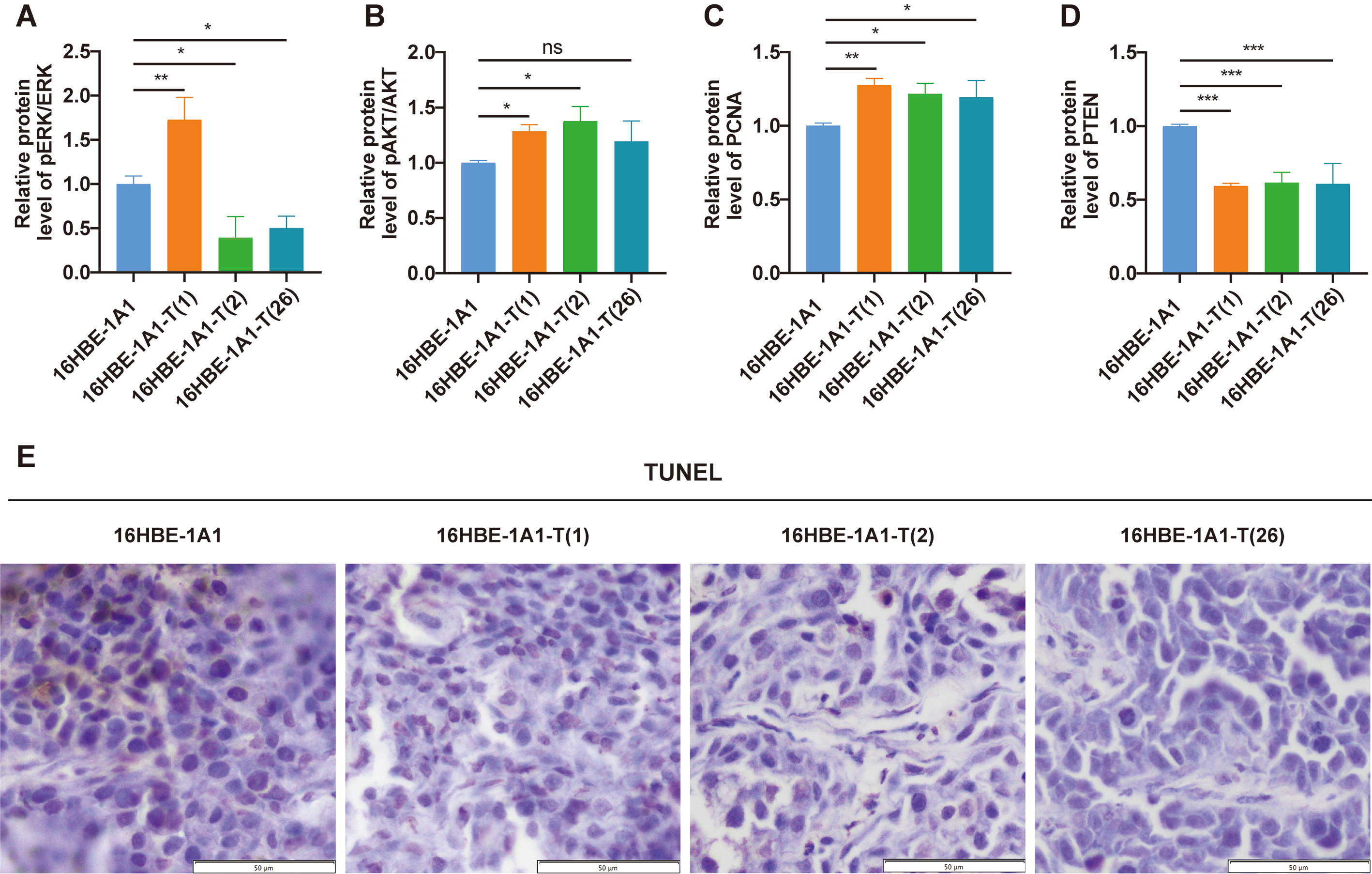


**Figure S2.** **Effects of DEE-OEs-induced transformation cells on lung cancer-related protein expression** **and TUNEL staining in xenografts. A.** Quantitative analysis of western blot showing the expression levels of pERK/ERK in 16HBE-1A1 cells and three clonal cell lines. **B.** Quantitative analysis of western blot showing the expression levels of pAKT/AKT in 16HBE-1A1 cells and three clonal cell lines. **C.** Quantitative analysis of western blot showing the expression levels of PCNA in 16HBE-1A1 cells and three clonal cell lines. **D.** Quantitative analysis of western blot showing the expression levels of PTEN in 16HBE-1A1 cells and three clonal cell lines. **E.** Representative TUNEL staining of xenografts derived from 16HBE-1A1 cells and three clonal cell lines. Scale bar = 50 μM.


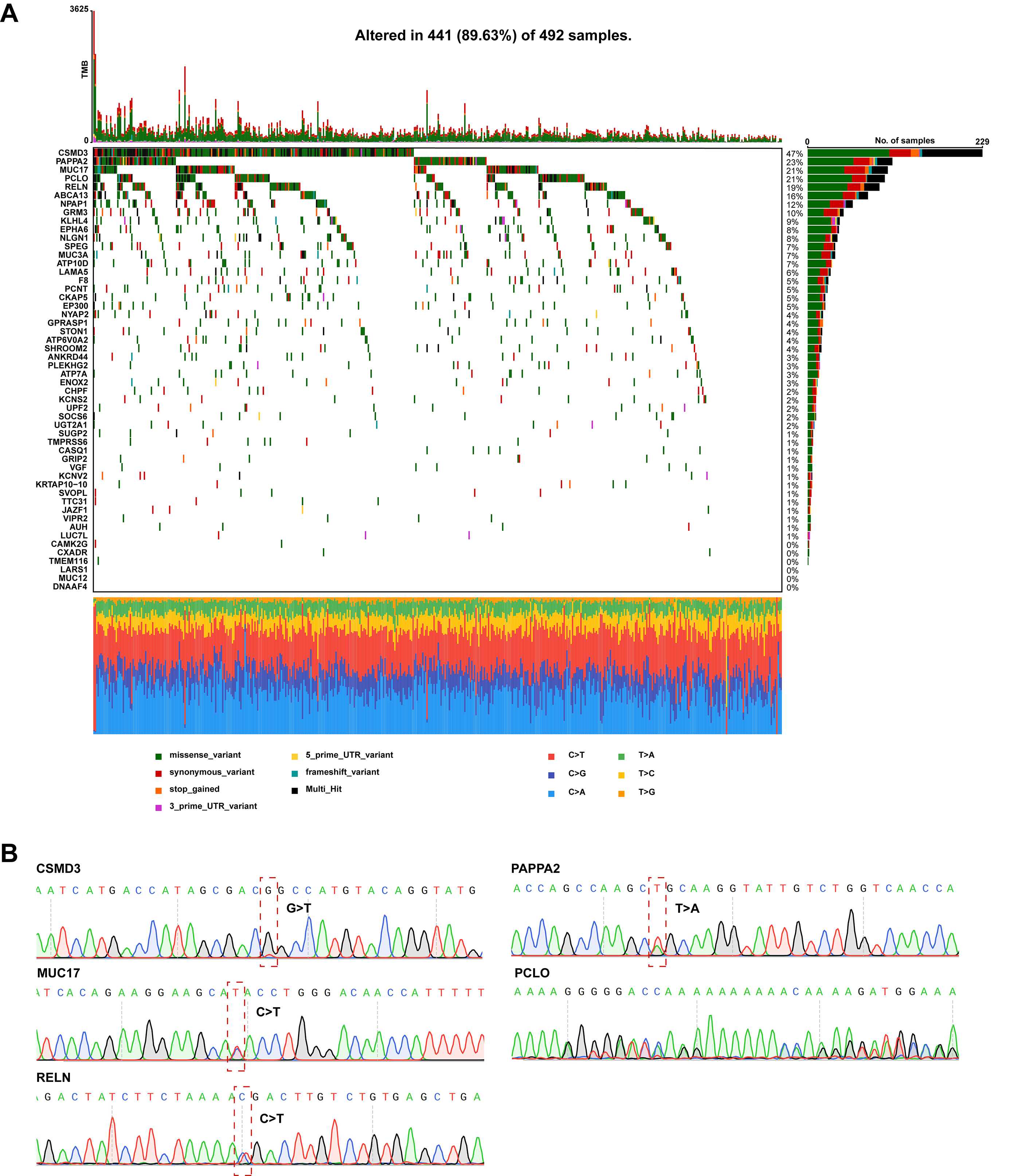


**Figure S3.** **Mutation landscape of 52 exon-region mutant genes induced by DEE-OEs exposure in TCGA-LUSC.** **A.** Mutation frequency and types of 52 exon-region mutated genes in TCGA-LUSC. Each row represents a gene, sorted by mutation frequency from high to low, and each column represents a sample (total of 492 cases). **B.** Sanger sequencing of CSMD3, PAPPA2, MUC17, PCLO, and RELN.


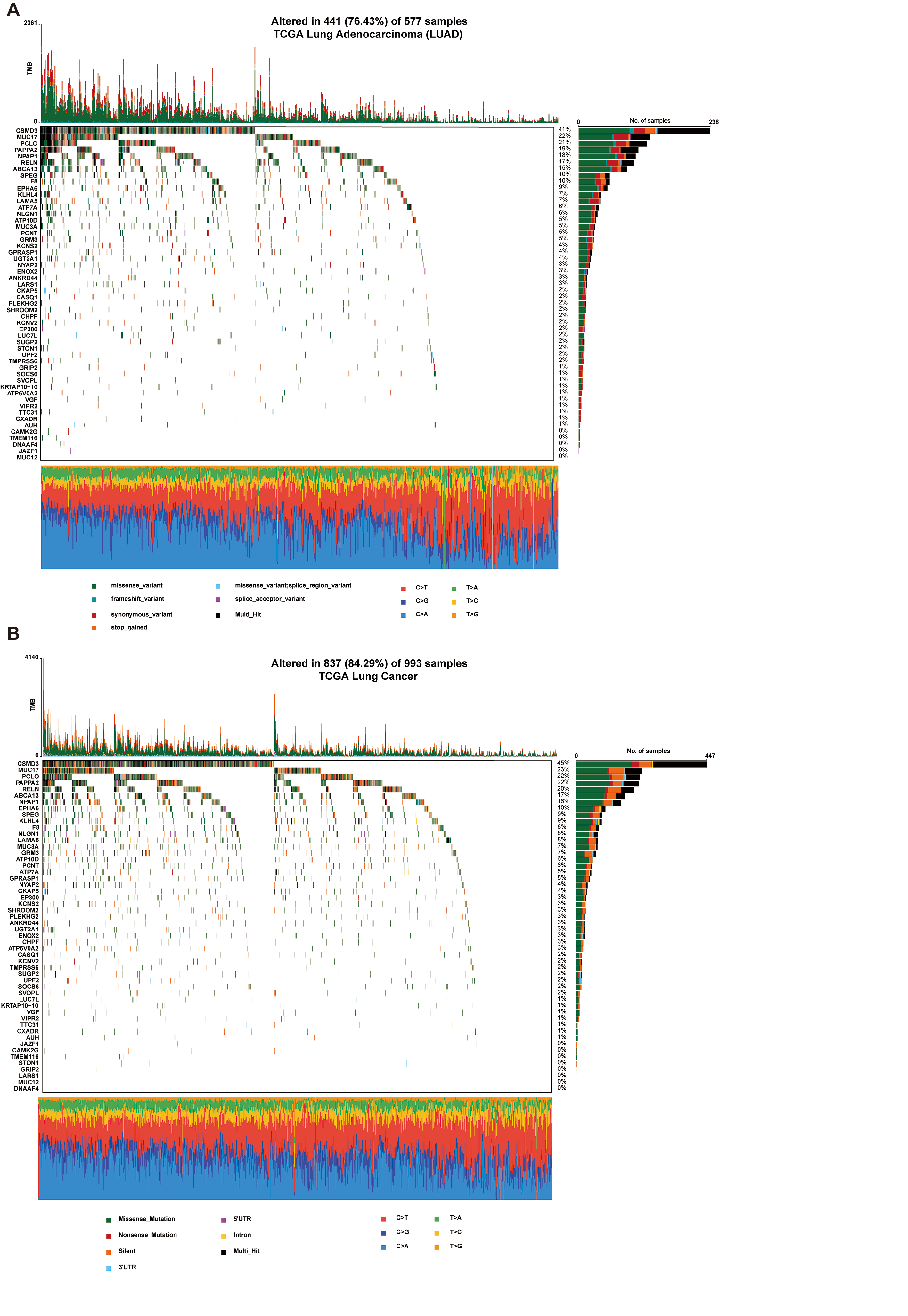


**Figure S4.** **Mutation landscape of 52 exon-region mutant genes induced by DEE-OEs exposure in other lung cancer. A.** Mutation frequency and types of 52 exon-region mutated genes in TCGA-LUAD. **B.** Mutation frequency and types of 52 exon-region mutated genes in lung cancer. Each row represents a gene, sorted by mutation frequency from high to low, and each column represents a sample.


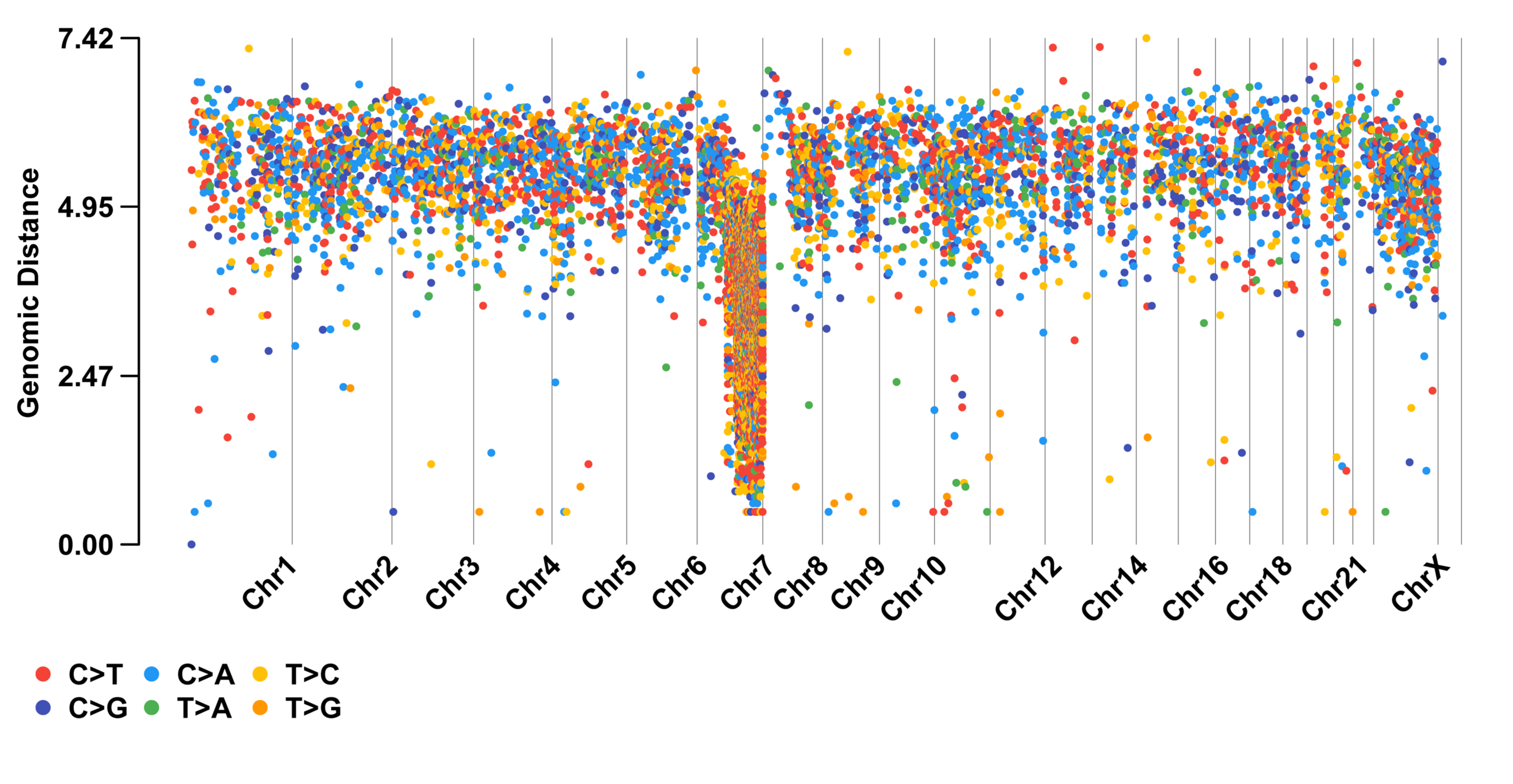


**Figure S5.** **Localized hypermutation rainfall plot induced by DEE-OEs exposure.** Rainfall plot, each dot represents a somatic mutation. x-axis shows the chromosomes of the human genome, while y-axis represents the genomic distance (in log scale) between each mutation and its nearby one.


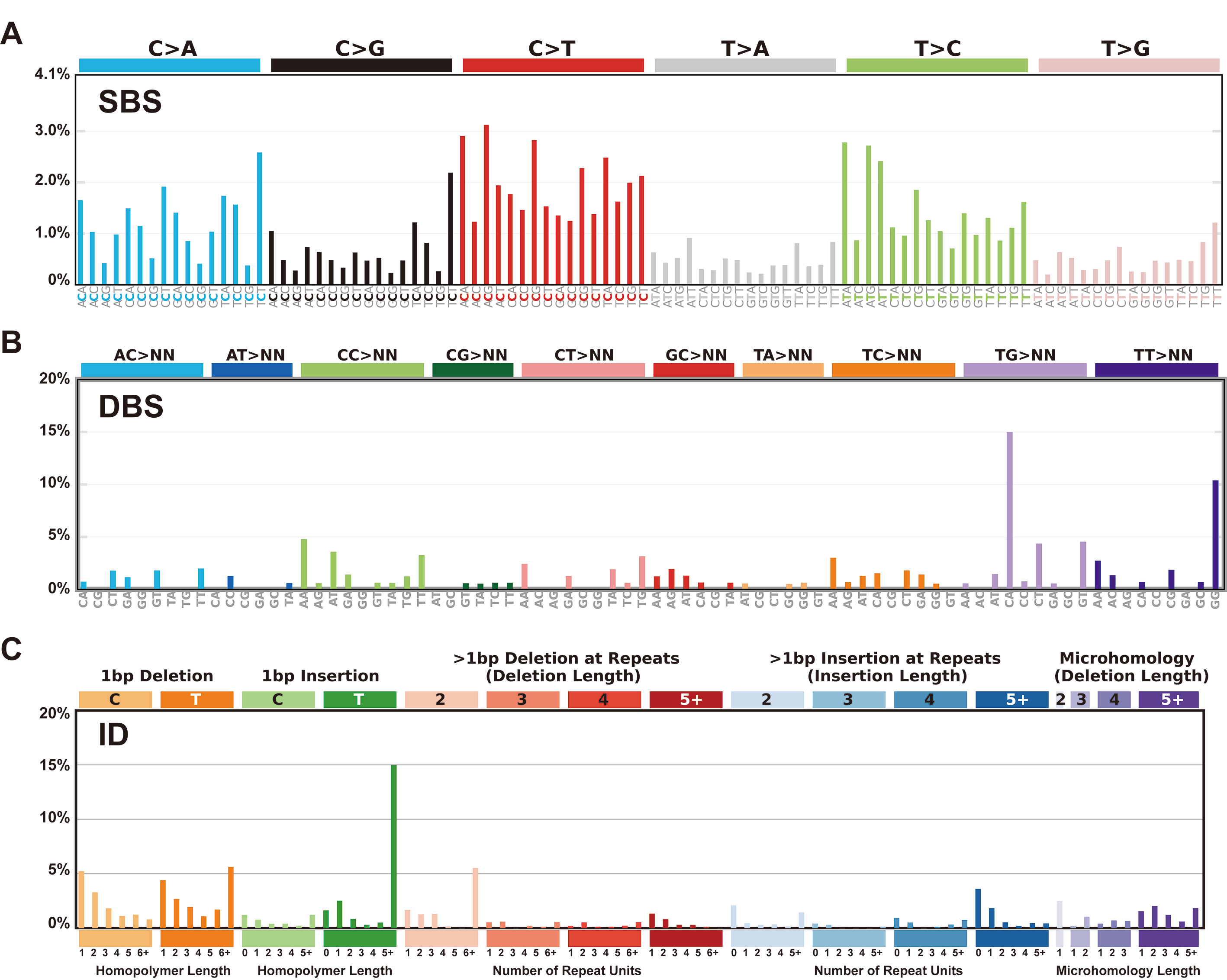


**Figure S6.** **De novo decomposition of mutational signatures in 16HBE-1A1-T cells.** **A.** SBS mutational signature in de novo decomposition in 16HBE-1A1-T cells. **B.** DBS mutational signature in de novo decomposition in 16HBE-1A1-T cells. **C.** ID mutational signature in de novo decomposition in 16HBE-1A1-T cells.

**
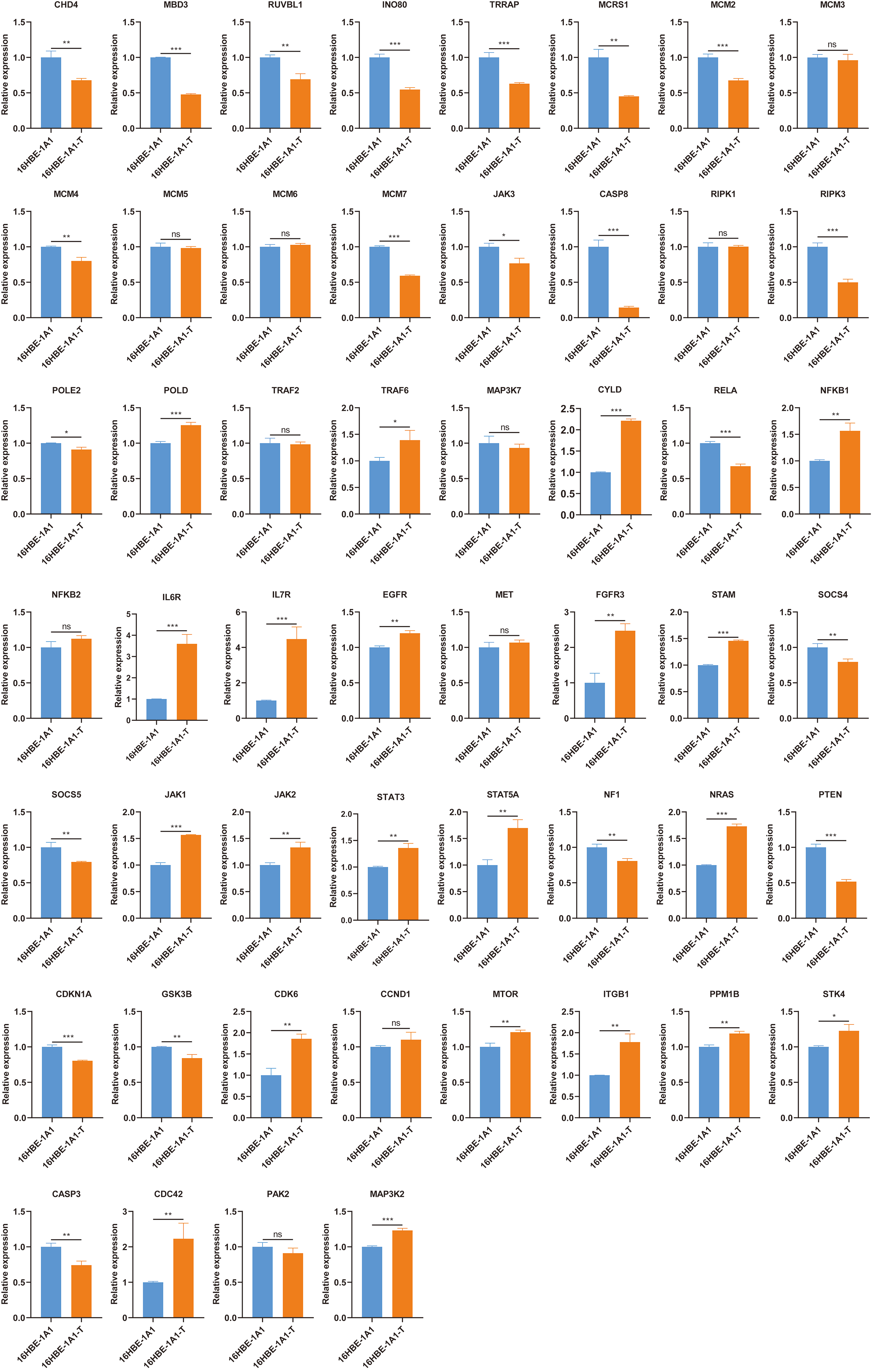
**

**Figure S7.** **DNA damage repair and lung cancer-related signaling pathways were verified by qPCR.** mRNA expression levels in 16HBE-1A1 and 16HBE-1A1-T cells were analyzed by Student’s *t*-test. **p* < 0.05, ***p* < 0.01, and ****p* < 0.001


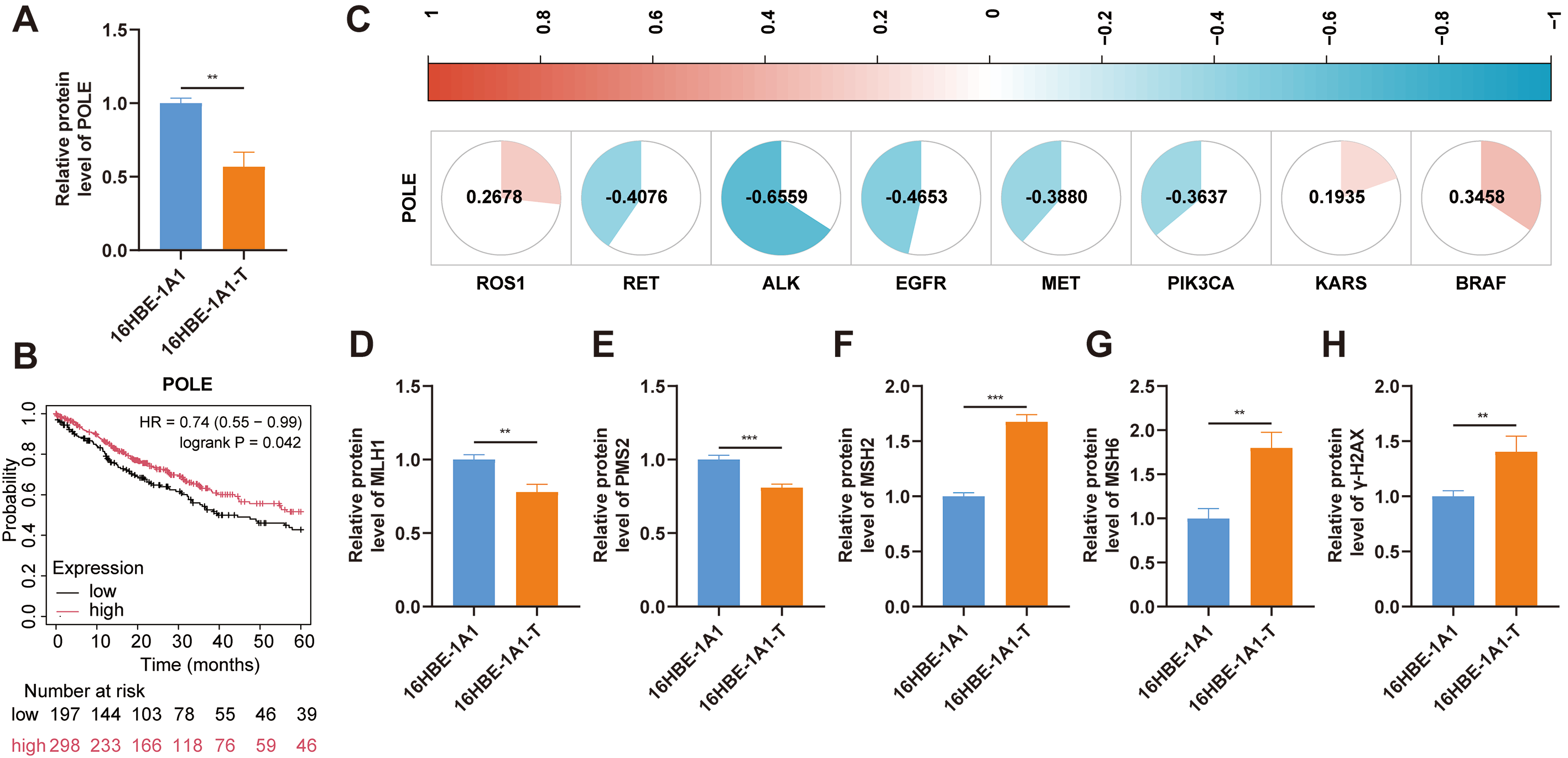


**Figure S8.** **DEE-OEs-induced malignant transformation cells exhibit POLE deficiency, MMR defects, and increased DNA damage. A.** Quantitative analysis of western blot showing the expression levels of POLE in 16HBE-1A1 and 16HBE-1A1-T cells. **B.** Using Kaplan-Meier Plotter, the correlation between POLE expression levels and overall survival in LUSC patients was analyzed, and survival curves were generated. **C.** Correlation analysis between POLE and driver genes in LUSC. **D, E, F, G.** Quantitative analysis of western blot showing the expression levels of MLH1, PMS2, MSH2, MSH6 in 16HBE-1A1 and 16HBE-1A1-T cells. **H.** Quantitative analysis of western blot showing the expression levels of γ-H2AX in 16HBE-1A1 and 16HBE-1A1-T cells.


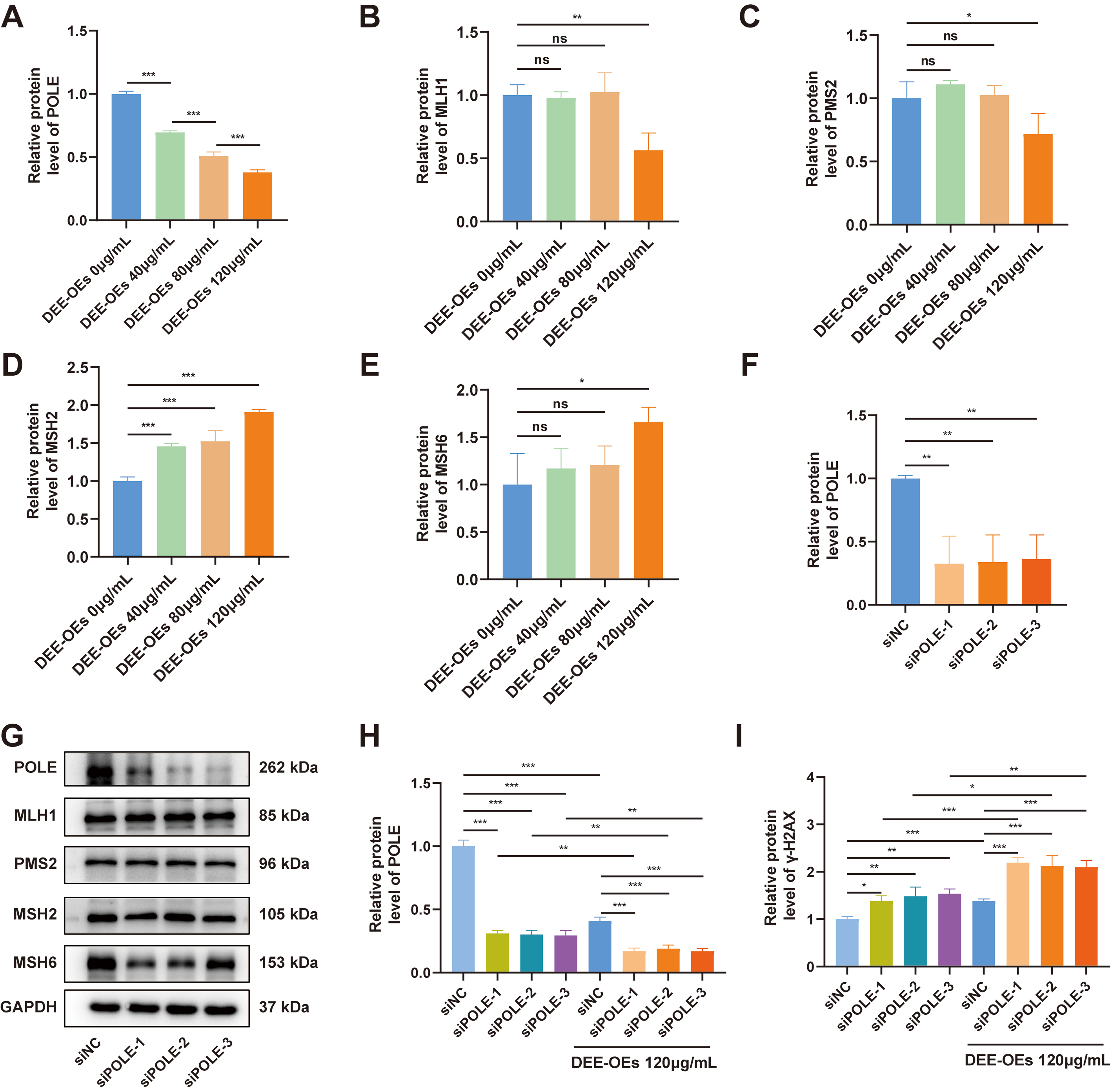


**Figure S9.** **Effects of DEE-OEs exposure and POLE knockdown on the protein expression of MMR and γ-H2AX. A.** Quantitative analysis of western blot results showing POLE expression levels after 20 weeks of exposure to 0, 40, 80, and 120 μg/mL DEE-OEs. **B, C, D, E.** Quantitative analysis of western blot results showing the expression levels of MLH1, PMS2, MSH2, and MSH6 after 20 weeks of exposure to 0, 40, 80, and 120 μg/mL DEE-OEs. **F.** Quantitative analysis of western blot results showing POLE expression levels following siRNA-mediated knockdown. **G.** Protein expression levels of MLH1, PMS2, MSH2, and MSH6 following POLE knockdown. **H, I.** Quantitative analysis of western blot results showing the expression levels of POLE and γ-H2AX in 16HBE-1A1 cells after POLE knockdown, DEE-OEs exposure, and combined treatment.


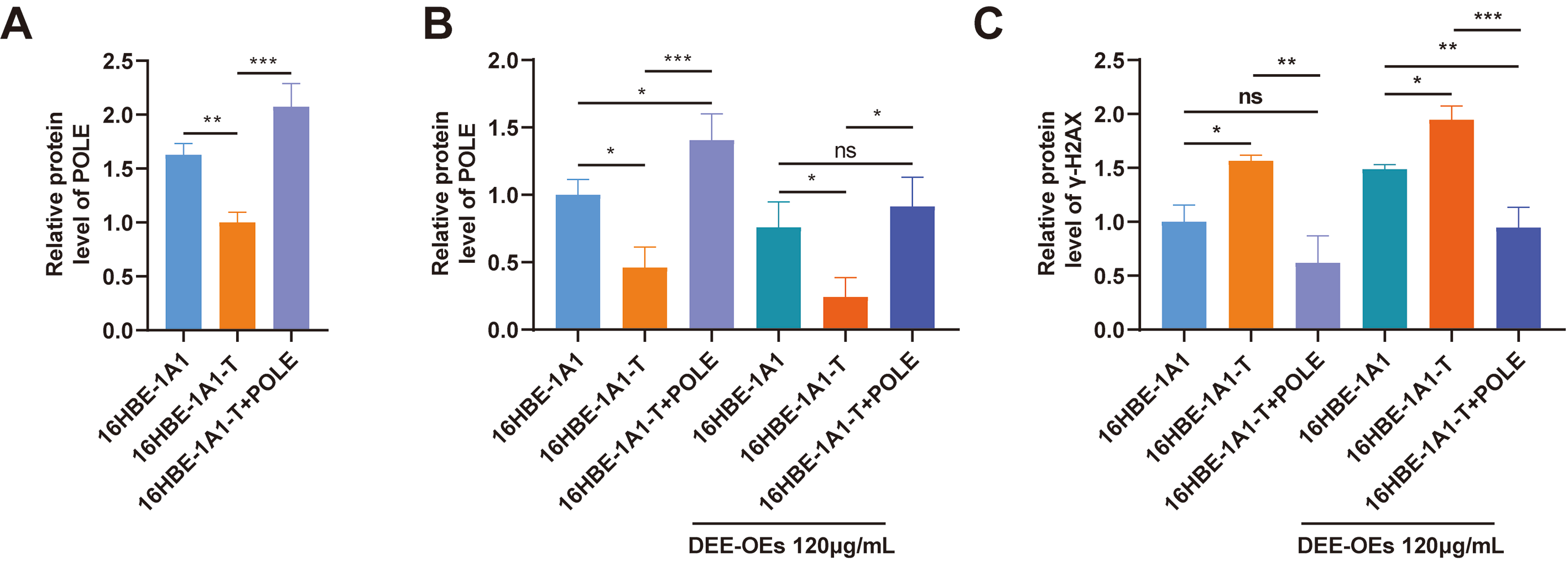


**Figure S10. Effects of DEE-OEs exposure and POLE overexpression on the protein expression of γ-H2AX. A.** Quantitative analysis of western blot results showing the levels of POLE overexpression. **B, C.** Quantitative analysis of western blot results showing the expression levels of POLE and γ-H2AX in 16HBE-1A1 cells following POLE overexpression, DEE-OEs exposure, and combined treatment.

**Table S1****. Major components and concentrations of PAHs in DEE**

| Components | Concentrations  [μg/m^3^] | | | TEFs | BaPeq  [μg/m^3^] |
| --- | --- | --- | --- | --- | --- |
| NAP | 6.824 | 9.889 | 8.28 | 0.001 | 0.008331 |
| ACY | 0.177 | 0.204 | 0.208 | 0.001 | 0.000196 |
| FLR | 0.567 | 0.641 | 0.44 | 0.001 | 0.000549 |
| PHE | 3.732 | 3.657 | 3.431 | 0.001 | 0.003607 |
| ANT | 0.525 | 0.487 | 0.405 | 0.01 | 0.004723 |
| FLT | 3.621 | 0.33 | 0.276 | 0.001 | 0.001409 |
| PYR | 16.12 | 14.205 | 13.105 | 0.001 | 0.014477 |
| BaA | 1.166 | 0.977 | 1.117 | 0.1 | 0.108667 |
| CHR | 2.401 | 2.407 | 2.252 | 0.01 | 0.023533 |
| BbF | 0.805 | 0.957 | 0.751 | 0.1 | 0.083767 |
| BaP | 0.357 | 0.468 | 0.347 | 1 | 0.390667 |
| IP | 0.141 | 0.129 | 0.114 | 0.1 | 0.0128 |
| BghiP | 0.131 | 0.153 | 0.16 | 0.01 | 0.00148 |
| Total |  |  |  |  | 0.654206 |

**Table S2. Somatic mutations in exonic regions identified by WGS**

| Chromosome | Start | End | Ref. | Alt. | Gene | Mutation type |
| --- | --- | --- | --- | --- | --- | --- |
| chr1 | 160192833 | 160192837 | TTGGC | - | CASQ1 | Frame_Shift_Del |
| chr1 | 176769779 | 176769779 | T | A | PAPPA2 | Missense_Mutation |
| chr2 | 74491548 | 74491548 | G | A | TTC31 | Missense_Mutation |
| chr2 | 197013633 | 197013633 | C | A | ANKRD44 | Missense_Mutation |
| chr2 | 219473834 | 219473834 | G | T | SPEG | Missense_Mutation |
| chr2 | 219543528 | 219543528 | G | C | CHPF | Missense_Mutation |
| chr2 | 225582326 | 225582326 | C | A | NYAP2 | Missense_Mutation |
| chr3 | 14507677 | 14507677 | - | C | GRIP2 | Frame_Shift_Ins |
| chr3 | 97637985 | 97637985 | G | A | EPHA6 | Missense_Mutation |
| chr3 | 174279034 | 174279034 | C | G | NLGN1 | Missense_Mutation |
| chr4 | 69639141 | 69639141 | A | G | UGT2A1 | Missense_Mutation |
| chr5 | 146164387 | 146164387 | T | C | LARS1 | Missense_Mutation |
| chr7 | 27832894 | 27832894 | C | T | JAZF1 | Missense_Mutation |
| chr7 | 48506378 | 48506378 | A | C | ABCA13 | Missense_Mutation |
| chr7 | 82760738 | 82760738 | - | T | PCLO | Frame_Shift_Ins |
| chr7 | 100957210 | 100957210 | A | G | MUC3A | Missense_Mutation |
| chr7 | 100993960 | 100993960 | C | T | MUC12 | Missense_Mutation |
| chr7 | 101001098 | 101001098 | C | T | MUC12 | Missense_Mutation |
| chr7 | 101043217 | 101043217 | C | T | MUC17 | Missense_Mutation |
| chr7 | 101163805 | 101163805 | C | G | VGF | Missense_Mutation |
| chr7 | 103697921 | 103697921 | C | T | RELN | Missense_Mutation |
| chr7 | 138649040 | 138649040 | G | A | SVOPL | Missense_Mutation |
| chr7 | 159096875 | 159096875 | A | G | VIPR2 | Missense_Mutation |
| chr8 | 98428945 | 98428945 | G | C | KCNS2 | Missense_Mutation |
| chr8 | 112650296 | 112650296 | G | T | CSMD3 | Missense_Mutation |
| chr9 | 2718712 | 2718712 | C | T | KCNV2 | Missense_Mutation |
| chr9 | 91355942 | 91355942 | G | T | AUH | Missense_Mutation |
| chr10 | 11979070 | 11979070 | - | C | UPF2 | Frame_Shift_Ins |
| chr10 | 73817115 | 73817115 | T | C | CAMK2G | Missense_Mutation |
| chr11 | 46744156 | 46744156 | C | A | CKAP5 | Missense_Mutation |
| chr12 | 111991871 | 111991884 | GATAAAAAAGTGGT | - | TMEM116 | Frame_Shift_Del |
| chr15 | 24677279 | 24677279 | C | A | NPAP1 | Missense_Mutation |
| chr16 | 190553 | 190553 | C | G | LUC7L | Missense_Mutation |
| chr19 | 18994464 | 18994464 | C | A | SUGP2 | Missense_Mutation |
| chr21 | 17559098 | 17559098 | G | T | CXADR | Missense_Mutation |
| chr21 | 44637521 | 44637521 | C | A | KRTAP10-10 | Missense_Mutation |
| chr21 | 46397405 | 46397405 | T | G | PCNT | Missense_Mutation |
| chr21 | 46397407 | 46397407 | T | G | PCNT | Missense_Mutation |
| chr22 | 37084369 | 37084369 | A | - | TMPRSS6 | Frame_Shift_Del |
| chr22 | 41178573 | 41178573 | C | G | EP300 | Missense_Mutation |
| chrX | 78033635 | 78033635 | A | G | ATP7A | Missense_Mutation |
| chrX | 87633878 | 87633878 | G | C | KLHL4 | Missense_Mutation |
| chrX | 102654797 | 102654797 | C | T | GPRASP1 | Missense_Mutation |
| chrX | 154896097 | 154896097 | T | G | F8 | Missense_Mutation |
| chr2 | 48581162 | 48581162 | C | T | STON1 | Nonsense_Mutation |
| chr4 | 47572219 | 47572219 | G | T | ATP10D | Nonsense_Mutation |
| chr7 | 86786560 | 86786560 | C | A | GRM3 | Nonsense_Mutation |
| chr12 | 123744305 | 123744305 | G | T | ATP6V0A2 | Nonsense_Mutation |
| chr15 | 55439512 | 55439512 | C | A | DNAAF4 | Nonsense_Mutation |
| chr18 | 70324780 | 70324780 | A | T | SOCS6 | Nonsense_Mutation |
| chr19 | 39423155 | 39423155 | G | T | PLEKHG2 | Nonsense_Mutation |
| chr20 | 62327922 | 62327922 | C | A | LAMA5 | Nonsense_Mutation |
| chrX | 9937282 | 9937282 | C | T | SHROOM2 | Nonsense_Mutation |
| chrX | 130667651 | 130667652 | GG | TT | ENOX2 | Nonsense_Mutation |

**Table S3. Statistically significant pathways**

| ID | Description | SetSize | EnrichmentScore | NES | *p*-value | qvalue |
| --- | --- | --- | --- | --- | --- | --- |
| hsa05322 | Systemic lupus erythematosus | 71 | -0.8215 | -3.1205 | 1.00E-10 | 9.16E-09 |
| hsa04613 | Neutrophil extracellular trap formation | 127 | -0.59964 | -2.505 | 1.00E-10 | 9.16E-09 |
| hsa05034 | Alcoholism | 135 | -0.56506 | -2.38757 | 1.00E-10 | 9.16E-09 |
| hsa03082 | ATP-dependent chromatin remodeling | 104 | -0.41816 | -1.70786 | 0.001668 | 0.024146 |
| hsa04350 | TGF-beta signaling pathway | 76 | -0.43734 | -1.68283 | 0.003769 | 0.038352 |
| hsa05144 | Malaria | 21 | -0.55356 | -1.61871 | 0.022809 | 0.12392 |
| hsa03030 | DNA replication | 35 | -0.46546 | -1.54657 | 0.028986 | 0.144752 |
| hsa05203 | Viral carcinogenesis | 169 | -0.3501 | -1.50881 | 0.003729 | 0.038352 |
| hsa04080 | Neuroactive ligand-receptor interaction | 95 | -0.37517 | -1.50337 | 0.013517 | 0.088421 |
| hsa00650 | Butanoate metabolism | 18 | -0.52437 | -1.47226 | 0.057582 | 0.232644 |
| hsa03010 | Ribosome | 128 | -0.35056 | -1.4683 | 0.015552 | 0.096033 |
| hsa04390 | Hippo signaling pathway | 128 | -0.34617 | -1.44993 | 0.019665 | 0.112556 |
| hsa04217 | Necroptosis | 115 | -0.33565 | -1.38273 | 0.029505 | 0.144752 |
| hsa04670 | Leukocyte transendothelial migration | 80 | -0.35028 | -1.35864 | 0.056537 | 0.231833 |
| hsa04020 | Calcium signaling pathway | 130 | 0.297393 | 1.303277 | 0.050236 | 0.212333 |
| hsa04010 | MAPK signaling pathway | 216 | 0.278199 | 1.312128 | 0.025851 | 0.134006 |
| hsa05168 | Herpes simplex virus 1 infection | 376 | 0.26213 | 1.320805 | 0.006255 | 0.052144 |
| hsa05135 | Yersinia infection | 113 | 0.310674 | 1.339049 | 0.039112 | 0.170563 |
| hsa05120 | Epithelial cell signaling in Helicobacter pylori infection | 60 | 0.356853 | 1.368117 | 0.060325 | 0.240195 |
| hsa04611 | Platelet activation | 81 | 0.338491 | 1.379635 | 0.037239 | 0.165014 |
| hsa05200 | Pathways in cancer | 379 | 0.273466 | 1.379944 | 0.001617 | 0.024146 |
| hsa04810 | Regulation of actin cytoskeleton | 167 | 0.304389 | 1.381716 | 0.008926 | 0.067581 |
| hsa04145 | Phagosome | 94 | 0.328146 | 1.385913 | 0.056207 | 0.231833 |
| hsa03013 | Nucleocytoplasmic transport | 99 | 0.327075 | 1.387972 | 0.040164 | 0.172413 |
| hsa04072 | Phospholipase D signaling pathway | 99 | 0.330984 | 1.404558 | 0.034838 | 0.159519 |
| hsa04664 | Fc epsilon RI signaling pathway | 49 | 0.391286 | 1.435486 | 0.037067 | 0.165014 |
| hsa04064 | NF-kappa B signaling pathway | 72 | 0.363189 | 1.453532 | 0.03138 | 0.148643 |
| hsa04926 | Relaxin signaling pathway | 94 | 0.34464 | 1.455578 | 0.030711 | 0.148024 |
| hsa01521 | EGFR tyrosine kinase inhibitor resistance | 66 | 0.371797 | 1.457092 | 0.023958 | 0.126579 |
| hsa05132 | Salmonella infection | 208 | 0.312986 | 1.459714 | 0.002122 | 0.027759 |
| hsa05205 | Proteoglycans in cancer | 157 | 0.324176 | 1.462796 | 0.005762 | 0.051524 |
| hsa04668 | TNF signaling pathway | 95 | 0.347387 | 1.4689 | 0.012148 | 0.085574 |
| hsa05417 | Lipid and atherosclerosis | 150 | 0.330366 | 1.474089 | 0.005196 | 0.049222 |
| hsa04730 | Long-term depression | 36 | 0.428399 | 1.47748 | 0.034142 | 0.158984 |
| hsa04151 | PI3K-Akt signaling pathway | 234 | 0.314468 | 1.499496 | 0.001279 | 0.021962 |
| hsa04510 | Focal adhesion | 151 | 0.336512 | 1.503658 | 0.003188 | 0.038085 |
| hsa04137 | Mitophagy - animal | 67 | 0.382559 | 1.508597 | 0.016267 | 0.097155 |
| hsa04630 | JAK-STAT signaling pathway | 96 | 0.360467 | 1.518389 | 0.008304 | 0.065184 |
| hsa04261 | Adrenergic signaling in cardiomyocytes | 88 | 0.369136 | 1.524325 | 0.009109 | 0.067581 |
| hsa04726 | Serotonergic synapse | 54 | 0.411658 | 1.541985 | 0.020449 | 0.114654 |
| hsa04971 | Gastric acid secretion | 44 | 0.428781 | 1.547044 | 0.01573 | 0.096033 |
| hsa04720 | Long-term potentiation | 45 | 0.42971 | 1.555391 | 0.012664 | 0.086985 |
| hsa04918 | Thyroid hormone synthesis | 42 | 0.434893 | 1.559321 | 0.023003 | 0.12392 |
| hsa04014 | Ras signaling pathway | 154 | 0.348521 | 1.562448 | 0.00184 | 0.025279 |
| hsa04071 | Sphingolipid signaling pathway | 98 | 0.371114 | 1.568088 | 0.004053 | 0.039768 |
| hsa04750 | Inflammatory mediator regulation of TRP channels | 68 | 0.398451 | 1.573152 | 0.01415 | 0.090406 |
| hsa04621 | NOD-like receptor signaling pathway | 128 | 0.360961 | 1.585533 | 0.00167 | 0.024146 |
| hsa00510 | N-Glycan biosynthesis | 47 | 0.434006 | 1.585679 | 0.009347 | 0.067581 |
| hsa04140 | Autophagy - animal | 154 | 0.355033 | 1.591639 | 0.00108 | 0.019782 |
| hsa00983 | Drug metabolism - other enzymes | 38 | 0.458007 | 1.60056 | 0.019051 | 0.11136 |
| hsa04912 | GnRH signaling pathway | 70 | 0.404763 | 1.602502 | 0.005814 | 0.051524 |
| hsa04144 | Endocytosis | 217 | 0.340719 | 1.606367 | 0.00019 | 0.006527 |
| hsa05143 | African trypanosomiasis | 15 | 0.581223 | 1.617542 | 0.026716 | 0.135921 |
| hsa01250 | Biosynthesis of nucleotide sugars | 32 | 0.485893 | 1.618491 | 0.013022 | 0.08726 |
| hsa05163 | Human cytomegalovirus infection | 161 | 0.35953 | 1.627182 | 0.000289 | 0.007943 |
| hsa04120 | Ubiquitin mediated proteolysis | 134 | 0.371357 | 1.627636 | 0.000437 | 0.010916 |
| hsa04015 | Rap1 signaling pathway | 148 | 0.367 | 1.64028 | 0.000611 | 0.013982 |
| hsa00520 | Amino sugar and nucleotide sugar metabolism | 42 | 0.468811 | 1.680934 | 0.007297 | 0.058966 |
| hsa04933 | AGE-RAGE signaling pathway in diabetic complications | 79 | 0.426814 | 1.738397 | 0.001034 | 0.019782 |
| hsa04070 | Phosphatidylinositol signaling system | 81 | 0.426526 | 1.738452 | 0.000956 | 0.019782 |
| hsa04640 | Hematopoietic cell lineage | 30 | 0.537302 | 1.756827 | 0.003641 | 0.038352 |
| hsa04216 | Ferroptosis | 33 | 0.528505 | 1.788028 | 0.002268 | 0.028318 |
| hsa00534 | Glycosaminoglycan biosynthesis - heparan sulfate / heparin | 17 | 0.619389 | 1.790983 | 0.006263 | 0.052144 |
| hsa03060 | Protein export | 23 | 0.580967 | 1.799114 | 0.003544 | 0.038352 |
| hsa04540 | Gap junction | 58 | 0.488799 | 1.853446 | 0.000288 | 0.007943 |
| hsa00562 | Inositol phosphate metabolism | 63 | 0.487945 | 1.900566 | 0.000132 | 0.006066 |
| hsa04961 | Endocrine and other factor-regulated calcium reabsorption | 33 | 0.589 | 1.992695 | 0.000177 | 0.006527 |
| hsa04520 | Adherens junction | 82 | 0.494481 | 2.018718 | 6.18E-06 | 0.000339 |
| hsa04141 | Protein processing in endoplasmic reticulum | 152 | 0.463721 | 2.076541 | 4.71E-08 | 3.24E-06 |

**Table S4. WGS sequencing data quality assessment**

| **Sample** | **Flowcell/Lane** | **Raw reads** | **Raw data(G)** | **Effective(%)** | **Q20(%)** | **Q30(%)** | **GC Content(%)** |
| --- | --- | --- | --- | --- | --- | --- | --- |
| 16HBE-1A1 | HNTLTDSX5_L1 | 255,346,156 | 102.99 | 99.79 | 96.64 | 91.38 | 40.91 |
|  | HNTLHDSX5_L1 | 87,964,324 |  | 99.78 | 97.38 | 93.13 | 40.95 |
| 16HBE-1A1-T | HNTLTDSX5_L2 | 294,163,696 | 95.10 | 99.66 | 96.66 | 91.42 | 40.76 |
|  | HNTLHDSX5_L1 | 22,834,961 |  | 99.66 | 97.11 | 92.55 | 40.75 |

**Table S5. Primers used for PCR**

| **Gene** | **Forward primer** | **Reverse primer** |
| --- | --- | --- |
| CSMD3 | TGACCTGAATTTGATCCTTACTGG | CAAATGAGTTATTACCATCACAGGT |
| MUC17 | CAAAGCCGGTTCATTCTCC | GTAGTCATTGCGGGTGTTGT |
| PAPPA2 | GGCCTGAAGAGGGAGAAAG | AGTTGGGGACAAAGAGTGGT |
| PCLO | TCGTTTACAATCTGCTGGCT | CAGGGTAGAGAAGGCTTGGC |
| RELN | ACGCCAAAGGGGAGAATGTC | TCTGGTCCTTTAATAGTGGTTTTGG |

**Table S6. RNA-Seq sequencing data quality assessment**

| **Sample** | **Raw Reads** | **Clean Reads** | **Raw Bases(G)** | **Clean Bases(G)** | **Q20(%)** | **Q30(%)** | **GC Content(%)** |
| --- | --- | --- | --- | --- | --- | --- | --- |
| 16HBE-1A1-1 | 186209152 | 124088510 | 27.93 | 18.61 | 98.98 | 96.10 | 48.84 |
| 16HBE-1A1-2 | 176568450 | 172465986 | 26.49 | 23.36 | 98.38 | 94.76 | 49.66 |
| 16HBE-1A1-3 | 172023034 | 169095486 | 25.80 | 22.88 | 98.54 | 95.12 | 48.54 |
| 16HBE-1A1-T-1 | 208134576 | 203910472 | 31.22 | 27.68 | 98.44 | 94.78 | 47.28 |
| 16HBE-1A1-T-2 | 178338266 | 174531990 | 26.75 | 23.75 | 98.51 | 95.04 | 47.78 |
| 16HBE-1A1-T-3 | 198540552 | 194622142 | 29.78 | 26.22 | 98.50 | 94.94 | 47.48 |

**Table S7. Primers used for quantitative qRT-PCR**

| **Gene** | **Forward primer** | **Reverse primer** |
| --- | --- | --- |
| CASP3 | GGAAGCGAATCAATGGACTCTGG | GCATCGACATCTGTACCAGACC |
| CASP8 | AGAAGAGGGTCATCCTGGGAGA | TCAGGACTTCCTTCAAGGCTGC |
| CCND1 | TCTACACCGACAACTCCATCCG | TCTGGCATTTTGGAGAGGAAGTG |
| CDC42 | TGACAGATTACGACCGCTGAGTT | GGAGTCTTTGGACAGTGGTGAG |
| CDK6 | GGATAAAGTTCCAGAGCCTGGAG | GCGATGCACTACTCGGTGTGAA |
| CDKN1A | AGGTGGACCTGGAGACTCTCAG | TCCTCTTGGAGAAGATCAGCCG |
| CHD4 | CTGTTGCTGACTGGGACACCAT | TGGTCCTCCTTGGCAATGTCAG |
| CYLD | GGTAATCCGTTGGATCGGTCAG | AGTGCCTCTGAAGGTTCCATCC |
| EGFR | AACACCCTGGTCTGGAAGTACG | TCGTTGGACAGCCTTCAAGACC |
| FGFR3 | TCCATCTCCTGGCTGAAGAACG | TGTTCTCCACGACGCAGGTGTA |
| GAPDH | GTCTCCTCTGACTTCAACAGCG | ACCACCCTGTTGCTGTAGCCAA |
| GSK3B | CCGACTAACACCACTGGAAGCT | AGGATGGTAGCCAGAGGTGGAT |
| IL6R | GACTGTGCACTTGCTGGTGGAT | ACTTCCTCACCAAGAGCACAGC |
| IL7R | ATCGCAGCACTCACTGACCTGT | TCAGGCACTTTACCTCCACGAG |
| INO80C | CCACCAGAGATACCTGAGGAAC | CAACCTGGTCTGAGTAGCCACT |
| ITGB1 | GGATTCTCCAGAAGGTGGTTTCG | TGCCACCAAGTTTCCCATCTCC |
| JAK1 | GAGACAGGTCTCCCACAAACAC | GTGGTAAGGACATCGCTTTTCCG |
| JAK2 | CCAGATGGAAACTGTTCGCTCAG | GAGGTTGGTACATCAGAAACACC |
| JAK3 | AGTGACCCTCACTTCCTGCTGT | GGCTGAACCAAGGATGATGTGG |
| MAP3K2 | TACACCCGTCAGATTCTGGAGG | ATGGTCTGAAGCCGTTTGCTGG |
| MAP3K7 | CAGAGCAACTCTGCCACCAGTA | CATTTGTGGCAGGAACTTGCTCC |
| MBD3 | TACGACTCCTCCAACCAGGTCA | GTCGCTCTTGACCTTGTTGCTG |
| MCM2 | TGCCAGCATTGCTCCTTCCATC | AAACTGCGACTTCGCTGTGCCA |
| MCM3 | CGAGACCTAGAAAATGGCAGCC | GCAGTGCAAAGCACATACCGCA |
| MCM4 | CTTGCTTCAGCCTTGGCTCCAA | GTCGCCACACAGCAAGATGTTG |
| MCM5 | GACTTACTCGCCGAGGAGACAT | TGCTGCCTTTCCCAGACGTGTA |
| MCM6 | GACAACAGGAGAAGGGACCTCT | GGACGCTTTACCACTGGTGTAG |
| MCM7 | GCCAAGTCTCAGCTCCTGTCAT | CCTCTAAGGTCAGTTCTCCACTC |
| MCRS1 | GCAGTATTACCTGCTGGAGGAC | TGTTCCAGGACCTCATCTCGCA |
| MET | TGCACAGTTGGTCCTGCCATGA | CAGCCATAGGACCGTATTTCGG |
| MLH1 | GCCTTGGCACAGCATCAAACCA | ATGGCAAGGTCAAAGAGCGGTG |
| MSH2 | CAGCAGTCAGAGCCCTTAACCT | GAGAGGCTGCTTAATCCACTGG |
| MSH6 | AAGGACTGGCAGTCTGCTGTAG | CGGCAACAGAATTACTGGGCGA |
| MTOR | AGCATCGGATGCTTAGGAGTGG | CAGCCAGTCATCTTTGGAGACC |
| NF1 | GCCTTGAGGAAAACCAGCGGAA | TCCTACTGCACCGATGCTGTTC |
| NFKB1 | GCAGCACTACTTCTTGACCACC | TCTGCTCCTGAGCATTGACGTC |
| NFKB2 | GGCAGACCAGTGTCATTGAGCA | CAGCAGAAAGCTCACCACACTC |
| NRAS | GAAACCTCAGCCAAGACCAGAC | GGCAATCCCATACAACCCTGAG |
| PAK2 | CGACTCCAACACAGTGAAGCAG | TCACTACTGCGGGTGCTTCTGT |
| PMS2 | TATCGGCTCTGTGTTTGGGCAG | AGCATCGGAACAGCTCAAACCG |
| POLD1 | ACTACACGGGAGCCACTGTCAT | GCGTGGTGTAACACAGGTTGTG |
| POLE | ACGCTGGAAGAGGTGTATGGCT | GGAACGGTTCTCAGAGATGAGC |
| POLE2 | TGCGTCCGTTTTCCTAGCAGCA | GGGCAGACATAAAGAGGTAGGG |
| PPM1B | GGCTTGAGGTATCTGATGACCTG | CACCGCTTCATCTGAGACCTTG |
| PTEN | TGAGTTCCCTCAGCCGTTACCT | GAGGTTTCCTCTGGTCCTGGTA |
| RELA | TGAACCGAAACTCTGGCAGCTG | CATCAGCTTGCGAAAAGGAGCC |
| RIPK1 | TATCCCAGTGCCTGAGACCAAC | GTAGGCTCCAATCTGAATGCCAG |
| RIPK3 | GCTACGATGTGGCGGTCAAGAT | TTGGTCCCAGTTCACCTTCTCG |
| RUVBL1 | GAAGACAGAGGTGCTGATGGAG | CTCTGTCTCACACGGAGTTAGC |
| SOCS4 | GGGTAAGCACAGACTTGTCTCAG | TCACAGAGCCAGTCATAGGACC |
| SOCS5 | TCTGGAGACAGCCATACCCATG | GCTTCATAACGGTCCATCACTCC |
| STAM | GACCAGTTGCTACAGATGCTGC | ATGAGAGGTCCCATCTGGTGAC |
| STAT3 | CTTTGAGACCGAGGTGTATCACC | GGTCAGCATGTTGTACCACAGG |
| STAT5A | GTTCAGTGTTGGCAGCAATGAGC | AGCACAGTAGCCGTGGCATTGT |
| STK4 | CTGTGTAGCAGACATCTGGTCC | CTGGTTTTCGGAATGTGGGAGG |
| TRAF2 | GAGCAGAAGGTCTTGGAGATGG | GCAGACACATCTTGTAGCCGTAC |
| TRAF6 | CAATGCCAGCGTCCCTTCCAAA | CCAAAGGACAGTTCTGGTCATGG |
| TRRAP | GTGGACCTGTCTGAAGTCGTCA | TCACTTCCTGGGCAGAATCCAC |
